# Severe COVID-19: Drugs and Clinical Trials

**DOI:** 10.3390/jcm12082893

**Published:** 2023-04-16

**Authors:** Hazael Ramiro Ceja-Gálvez, Francisco Israel Renteria-Flores, Ferdinando Nicoletti, Jorge Hernández-Bello, Gabriela Macedo-Ojeda, José Francisco Muñoz-Valle

**Affiliations:** 1 Institute of Research in Biomedical Sciences, University Center of Health Sciences (CUCS), University of Guadalajara, Guadalajara 44340, Jalisco, Mexico; 2 Department of Biomedical and Biotechnological Sciences, University of Catania, 95123 Catania, Italy

**Keywords:** COVID-19 treatment, SARS-CoV-2, clinical trials, drugs COVID-19

## Abstract

By January of 2023, the COVID-19 pandemic had led to a reported total of 6,700,883 deaths and 662,631,114 cases worldwide. To date, there have been no effective therapies or standardized treatment schemes for this disease; therefore, the search for effective prophylactic and therapeutic strategies is a primary goal that must be addressed. This review aims to provide an analysis of the most efficient and promising therapies and drugs for the prevention and treatment of severe COVID-19, comparing their degree of success, scope, and limitations, with the aim of providing support to health professionals in choosing the best pharmacological approach. An investigation of the most promising and effective treatments against COVID-19 that are currently available was carried out by employing search terms including “Convalescent plasma therapy in COVID-19” or “Viral polymerase inhibitors” and “COVID-19” in the Clinicaltrials.gov and PubMed databases. From the current perspective and with the information available from the various clinical trials assessing the efficacy of different therapeutic options, we conclude that it is necessary to standardize certain variables—such as the viral clearance time, biomarkers associated with severity, hospital stay, requirement of invasive mechanical ventilation, and mortality rate—in order to facilitate verification of the efficacy of such treatments and to better assess the repeatability of the most effective and promising results.

## 1. Introduction

COVID-19 is an infectious disease that can generate severe flu-like symptoms with acute respiratory distress and an acute inflammatory state. Most patients present mild to moderate symptoms; however, 5–10% present severe states which can even lead to death [1,2,3]. By January of 2023, the COVID-19 pandemic had resulted in a reported total of 662,631,114 accumulated infections and 6,700,883 deaths [4]. It has also been reported that, in the United States and Germany, there have been 6.04 and 14.06 intensive care unit (ICU) admissions per million reported patients, respectively [5].

The primary pathology presented in critically ill patients is acute respiratory distress syndrome (ARDS) due to the occurrence of a cytokine storm, which involves dysregulation of the release of proinflammatory cytokines and chemokines, thus inducing an acute inflammatory state [6,7,8]. Multiple factors associated with severe COVID-19 and its mortality have also been reported, such as age older than 65 years, male sex, pre-existing comorbidities (e.g., hypertension, diabetes, cardiovascular disease, and chronic kidney disease), laboratory indices (e.g., C-reactive protein, CRP; lactate dehydrogenase, LDH; procalcitonin; aspartate aminotransferase, AST; and alanine aminotransferase, ALT), and proinflammatory cytokines (e.g., Interleukin-6, IL-6; IL-8; IL-10; IL-2R; and tumor necrosis factor, TNF-α) [9,10,11].

As this infection poses a significant public health problem, the search for effective prophylactic and therapeutic strategies is a primary goal that urgently needs to be ad-dressed.

**Figure 1:**
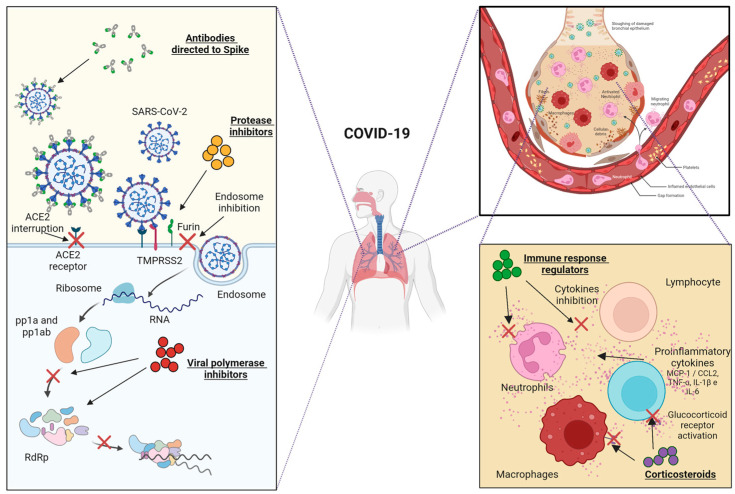
Primary mechanisms of potential COVID-19 drugs under development. The main mechanisms of action of the potential treatments against COVID-19 are schematized as follows: SARS-CoV-2 entry block (mediated by anti-Spike antibodies or protease inhibitors), replicative cycle arrest (mediated by viral polymerase inhibitors), and regulation of proinflammatory response (mediated by corticoids and other immune response regulators).

At present, the drugs designed to counter COVID-19 (Figure 1) present various different mechanisms of action. For example, viral polymerase inhibitors are nucleotide analogues that are incorporated into the nascent RNA and terminate its synthesis, limiting viral replication [12,13]. Protease inhibitors act through two mechanisms: they inhibit membrane proteases that facilitate viral entry into the cell, or inhibit proteases involved in the cleavage of the polypeptides (pp1a and pp1b) that make up the SARS-CoV-2 viral replication complex [14,15]. Corticosteroids are drugs that suppress the acute inflammatory response by inhibiting the expression of proinflammatory cytokines [16,17]. mAbs target the Spike protein, blocking the link between the viral receptor binding domain (RBD) and Angiotensin-converting enzyme 2 (ACE2), preventing viral internalization or viral opsonization by anti-Spike neutralizing antibodies (NAbs), leading to the formation of immune complexes (in collaboration with the complement) which are recognized by Fragment crystallizable (Fc) and complement receptors in antigen-presenting cells (APCs), stimulating their uptake by phagocytosis and thus triggering the immune response [18,19]. Immune response regulators are drugs capable of blocking the signal transduction of the main immune cells, targeting specific proteins and allosterically inhibiting crucial signaling pathways that trigger inflammatory responses. The cytosolic tails of cytokine and chemokine receptors are primarily affected [20,21].

**Figure 2:**
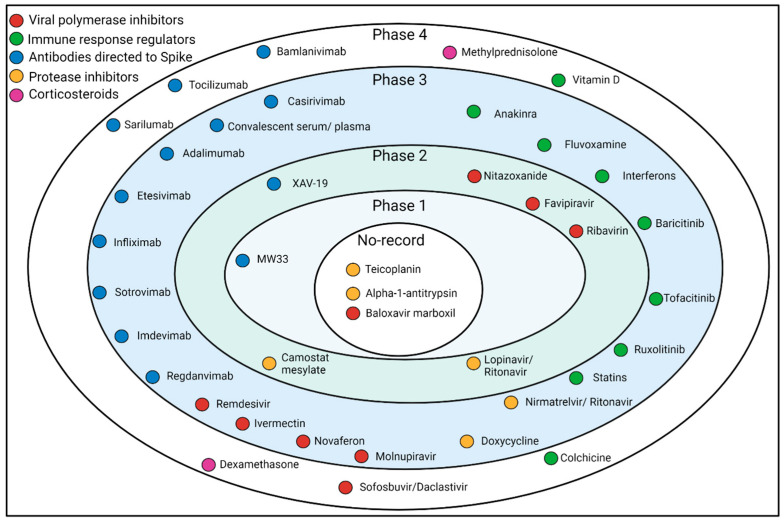
Phases of potential COVID-19 drugs in clinical trials. The main drugs with potential therapeutic use against severe COVID-19 and the clinical trial phases in which they are located are listed.

Considering the varied literature available on this topic, this comprehensive literature review describes the central action mechanisms of COVID-19 drugs by subgroup, including their targets and phase status in clinical trials (Figure 2). In addition, this review discusses the advantages and disadvantages of each of the drugs, as well as the challenges identified, in order to identify the more efficient therapeutic proposals.

## 2. Material and Methods

For this review, a literature search was performed in the PubMed and clinicaltrials.gov databases, considering publications from the beginning of the pandemic until 6 July 2022, using topics and subtopics related to use of viral polymerase inhibitors in COVID-19, convalescent plasma therapy in COVID-19, monoclonal antibodies in COVID-19, immune response regulators in COVID-19, and anti-inflammatory drugs for COVID-19. The reviewed publications included clinical trials in patients with severe COVID-19 and reported relevant results regarding mortality rate, hospital stay, ICU requirement, or laboratory parameters. The results were complemented with searches related to the specific background and mechanism of action for each selected drug.

## 3. Convalescent Plasma Therapy in COVID-19

Various studies have considered the possibility of using convalescent plasma (CP) in seriously ill patients with COVID-19 to increase the amount of neutralizing anti-SARS-CoV-2 antibodies, as other immune cells may confer better disease progression. CP therapy has been applied successfully to treat outbreaks of SARS, MERS, influenza H1N1, poliomyelitis, measles, and mumps with satisfactory efficacy and safety [22,23,24,25].

Immunotherapy with the polyclonal neutralizing antibodies present in CP has been shown to be safe and effective, as demonstrated in a study in which ten patients with severe COVID-19 illness were treated with 200 mL of CP (Titers 1:640) from recently recovered donors with previously detectable NAbs. A significant improvement in clinical symptoms and laboratory parameters was observed within three days after CP transfusion, compared to the control group. Other parameters tended to improve also, such as lymphocyte counts (0.65 × 109 per L in the treatment vs. 0.76 × 109 per L in the control group), decreases in C-reactive protein (18.13 mg/L vs. 55.98 mg/L), alanine aminotransferase (34.30 U/L vs. 42.00 U/L), and aspartate aminotransferase (30.30 U/L vs. 38.10 U/L), high level of neutralizing antibodies, the disappearance of viremia in 7 days, and decreased radiological images of lung lesions. A significant difference (*p* < 0.001) was found in survival between the treated and the control group. No severe adverse effects were detected after CP therapy. The improvement may be due to the effector mechanism of neutralizing antibodies that activate complement and phagocytosis. Further studies should be conducted to corroborate these findings in more significant cohorts [26].

In a clinical trial (NCT04338360) involving 5000 hospitalized patients (66% in the Intensive Care Unit, ICU) in the United States with a diagnosis of severe or life-threatening COVID-19, relatively low mortality (14.9%) was observed in treated patients at 7 days post-treatment, and the occurrence of adverse reactions was present in less than 1% (*n* = 36), including allergic reactions (*n* = 3) and mortality rate (0.3%). According to these results, CP transfusion may be safe in hospitalized patients with severe COVID-19 [27].

In a cohort study conducted in the Houston Methodist hospital with 316 COVID-19 patients, 136 were treated with CP (anti-RBD IgG titer of >1:1350) and 180 with conventional treatment.

The risk of overall mortality and mortality within 28 days in non-CP patients was significantly higher, compared with patients who received CP transfusion with an anti-RBD IgG titer of >1:1350 within 72 h of hospital admission (RR = 7.53; 95% CI: 1.12–50.46; *p* = 0.04; and RR = 5.92; 95% CI = 0.90–38.84; *p* = 0.06). According to the Kaplan–Meier test, at the 28-day follow-up, the control group had 7% overall mortality, in contrast with 1.2% overall mortality in the CP group, with a significant difference between groups (*p* = 0.047). This analysis suggests that transfusion of high anti-RBD IgG titer COVID-19 convalescent plasma early in hospitalization reduces severity and mortality in COVID-19 patients [28].

Earlier studies on CP therapy for MERS-CoV demonstrated that the minimal antibody titer to be effective should be higher than 1:80 to reach full viral clearance. The neutralizing antibody titer in recently recovered COVID-19 patients is above 1:640 [26]. This is encouraging as, based on the results related to MERS, there may be many potential convalescent plasma donors; however, it is essential to note that these SARS-CoV-2 antibodies decline rapidly over time. Therefore, further information is required to verify the optimal time range to consider someone as a donor for CP therapy [26]. Furthermore, while CP therapy for COVID-19 may provide a clinical benefit when it is given early in the course of the disease and when it contains high IgG titers, the optimal antibody titer for viral neutralization remains unknown; this must be addressed for future standardization of CP therapy protocols in clinical trials [27,29]. In a randomized controlled trial (NCT04348656) conducted in 921 COVID-19 hospitalized patients receiving oxygen therapy, 500 mL of CP did not reduce the risk of intubation or death at day 30. Intubation or death occurred in 32.4% of patients treated with CP, compared to 28% of the untreated group who received conventional care (RR = 1.16, 95% CI = 0.94–1.43, *p* = 0.18). The CP group had more serious adverse events than the control group that received standard treatment (33.4% vs. 26.4%; RR = 1.27, 95% CI = 1.02–1.57, *p* = 0.034). These data suggest that the transfusion of CP with unfavorable antibody profiles could be associated with worse clinical outcomes than standard care [30]. Unfavorable antibody profiles (measured by flow cytometry) can be defined as anti-Spike IgG antibodies directed to an epitope of the full Spike protein different than subunit 1, the presence of which in CP has been correlated with an increased risk of intubation or death. These non-functional anti-Spike IgG antibodies may be harmful due to their competition with functional anti-Spike Subunit 1 IgG antibodies, decreasing their ability to aggregate and crosslink Fraction Crystallized (Fc) receptors on target cells and, consequently, disrupting their neutralizing activity [30]. Similar observations have been made during human immune human immunodeficiency virus (HIV) vaccine trials, where the development of Immunoglobulin α (IgA) antibodies against the virus envelope paradoxically increased the risk of infection due to competition with IgG [31,32].

In another study in 526 COVID-19 hospitalized patients, there was no statistical difference in 28-day mortality between CP and control groups (25.5% vs. 27%, *p* = 0.06); nevertheless, 7-day mortality was statistically better for the CP group than the control group (9.1% vs. 19.8%, *p* = 0.01), as was found also at 14 days (14.8% vs. 23.6%, *p* = 0.01). After 72 h, transfusion resulted in a transition from the nasal cannula to room air (1 day vs. 4 days, *p* = 0.02). Moreover, the length of stay was longer in the CP group than in the control group (14.3 days vs. 11.4 days, *p* < 0.001). This study suggested that CP therapy among the hospitalized COVID-19 patients had only an immediate mortality benefit, but was not effective in the long-term [33].

The main concern regarding plasma transfusions from convalescent patients is transfusion-associated circulatory overload (TACO), which results in pulmonary edema and left atrial hypertension after circulatory overload and transfusion-related acute lung injury. The theoretical antibody-dependent enhancement (ADE) could deteriorate the clinical condition and induce an antibody-mediated proinflammatory effect [34,35,36,37]. Antibody administration to individuals with high viral loads may lead to the formation of immune complexes, which may contribute to proinflammatory immune responses through excessive complement activation [38].

Despite the above, preliminary results regarding CP transfusions in COVID-19 patients have indicated the apparent absence of antibody-related adverse effects, which could be explained by the preferential binding of the NAbs to the virus, rather than to immune cells or tissues that would be needed to enhance the proinflammatory immune responses responsible for adverse effects (e.g., ADE) [39].

Another study has indicated that antibody levels do not guarantee viral clearance, and transfusion of CP with high NAbs titers did not significantly modify viral load kinetics [39]. CP treatment in COVID-19 induced an early but transient effect on the antibody and cytokine profile of patients with severe disease and increased memory T and B lymphocytes at day 28 post-transfusion. At this date after treatment with CP, decreases in activated, effector, and effector memory CD4+ cells (*p* < 0.05) were observed, as well as reductions in activated and effector CD8+ (*p* < 0.01) T cells and naïve B cells (*p* = 0.001), compared with controls receiving conventional treatment. In contrast, increases in non-classical memory B cells (*p* < 0.0001) and central memory CD4+ T cells (*p* = 0.0252) were observed. Regarding the profile of proinflammatory cytokines, IL-6/IFN-γ (*p* = 0.0089) and IL-6/IL-10 (*p* = 0.0180) ratios were decreased in plasma recipients, compared to those who received standard therapy alone. These latter results may indicate the beneficial therapeutic implications of CP in COVID-19, thus justifying further studies [40].

In contrast to the favorable results obtained regarding the use of CP therapy in moderate to severe cases of COVID-19, in a previous systematic review assessing the efficacy and safety of CP therapy in 33 randomized controlled trials (9 single-center and 24 multi-center studies) that included 24,861 participants (of which 11,432 received CP therapy) concerning moderate to severe COVID-19, CP versus placebo or standard care alone did not reduce all-cause mortality risk at day 28 (risk ratio, RR: 0.98; 95% confidence interval, CI: 0.92–1.03; 220 per 1000; 21 randomized controlled trials, RCTs; 19,021 participants), had little or no impact on the need for invasive mechanical ventilation or death (RR: 1.03; 95% CI: 0.97–1.11; 296 per 1000; 6 RCTs; 14,477 participants) and, finally, had no impact on hospital stay (RR: 1.00; 95% CI: 0.97–1.02; 665 per 1000; 6 RCTs; 12,721 participants). The authors concluded that, according to the evidence, CP for individuals with moderate to severe disease does not reduce mortality and has little to no impact on clinical improvement, with the rate of worsened condition potentially being even higher [41].

Randomized controlled trials for CP therapy tend to present a high discrepancy in results, which may be due to the high methodological variability in the inclusion criteria, selection of appropriate donors, dosage, the concentration of neutralizing antibodies, and time of transfusion [42]. The clinical benefits of CP therapy need to be further investigated in randomized clinical studies considering the abovementioned variables. Therefore, the efficacy of CP therapy for COVID-19 remains unclear at present [30].

### Criteria to Consider for CP Therapy

Although CP therapy can provide polyclonal NAbs, which are able to opsonize viral particles, activate complement, and form immune complexes that will be taken up by APCs, thus triggering an effective immune response [43], there are several risks associated this therapy that must be considered, such as transmission of infectious diseases (e.g., HIV, hepatitis B, and hepatitis C; incidence one per two million transfusions in the U.S.) [44], allergic reactions (1–3% of transfusions) [45], transfusion-associated circulatory overload (TACO), transfusion-related acute lung injury (TRALI) [45], and the theoretical possibility of antibody-dependent enhancement (ADE) [46,47]. TACO is the leading cause of transfusion-related morbidity and mortality worldwide, occurring in 1–12% of individuals in at-risk populations [48]. The symptoms presented within 6 h of BT are acute respiratory distress, tachycardia, increased blood pressure, acute or worsening pulmonary edema, and evidence of a positive fluid balance [49]. The incidence of TRALI is 1 per 5000 transfusions, but it is of particular concern in severe COVID-19 due to potential priming of the pulmonary endothelium [50]. Plasma from a previously pregnant blood donor is a well-established risk factor for TRALI due to pregnancy-related alloimmunization; HLA antibody screening can mitigate this risk [51]. The role of CP in pregnancy and lactation requires further evaluation [52]. Special attention should be paid to the volume of CP administered to patients with risk factors for TACO, such as cardiorespiratory diseases, advanced age, renal impairment, and so on [49]. The passive immunization of patients with SARS-CoV-2 CP is theoretically beneficial in patients with end stage renal disease who are immunosuppressed and unable to mount an adequate immune response; however, according to preliminary results, CP does not appear to confer any clinical benefit in moderate-to-severe SARS-CoV-2 infected patients with chronic kidney disease on hemodialysis [53]. Further studies are required to assess the risks of CP therapy in other vulnerable groups.

## 4. Monoclonal Antibodies

Among the various potential therapeutic interventions used to treat or prevent COVID-19, one of the most successfully employed strategies is the use of monoclonal antibodies (mAbs). Some mAbs have demonstrated a high level of efficacy with a relative risk reduction in hospitalization and mortality (71.3% for casirivimab/imdevimab [54], 70% for bamlanivimab/etesevimab [55], and 85% for sotrovimab [56]), as well as an acceptable level of safety [57]. mAbs are one of the most promising therapeutic approaches in which the risk of an off-target effect is almost non-existent, due to their high specificity to the viral epitope or its target receptor, as in the case of targeting the Interleukin-6 receptor (IL-6R) [58].

Neutralizing antiviral mAbs have been successfully used to treat diverse pathologies such as Ebola (using mab114, REGN-EB3, and ZMapp) and respiratory syncytial virus (effectively treated with palivizumab) [59]. Such results have motivated search for and development of neutralizing mAbs for treatment and prevention in the context of the SARS-CoV-2 pandemic. At first, researchers and pharmaceutical companies focused on developing mAbs derived from convalescent patients. The methodology used for development mainly consists of isolating memory B lymphocytes specific to the SARS-CoV-2 Receptor Binding Domain (RBD), cloning, transfection and, finally, mAbs production. Several mAbs with neutralizing activity against SARS-CoV-2 have been discovered for use as potential treatment options; however, only a few of these are currently being tested in clinical trials [59].

According to preclinical studies, using a potent nMAb cocktail targeting the angiotensin-converting enzyme 2 (ACE2) and broad-nMAbs targeting of conserved regions within Spike may be effective for the treatment and prevention of COVID-19 [60]. The Spike protein has been identified as the major inducer of natural NAbs, mainly the RBD within the S1 unit, which can block the RBD–ACE2 interaction, although it has also been observed that some NAbs recognize epitopes on the S2 unit, which could also neutralize viral internalization [61,62,63]. Opsonization can prevent the binding between RBD and ACE2, mediating complement activation and phagocytosis by recognizing the Fc fraction and antibody-dependent cytotoxicity [64]. Some other NAbs are bound to viral proteins that are not essential for cell receptor recognition. However, their effector function lies in their capacity to exert conformational changes that do not allow for internalization of the virus within the cell membrane, or preventing the virion from merging its envelope with the endosome membrane, thus making it impossible to release the genetic material inside the infected cell [65,66].

### 4.1. mAbs Targeting SARS-CoV-2 Spike Protein

Due to their extraordinary antigen specificity, mAbs targeting the Spike protein of SARS-CoV-2—mainly aimed at RBD—could be some of the best candidates for neutralizing viral infection. Their high specificity can block the link between the viral RBD and ACE2, effectively preventing viral internalization [18]. The main disadvantage of their use is that the high specificity could be impaired by drastic changes in amino acids present in new SARS-CoV-2 variants, leading to non-recognition, ineffective opsonization, and evasion of neutralization [19].

Recent data have shown that, in newly emerging variants, several mutations can be identified in the S-RBD region, considerably reducing the mAbs neutralizing activity, representing an important challenge for mAbs therapy that threatens their protective efficacy against COVID-19. In this regard, the administration of mAb cocktails targeting different epitopes is considered as a strategy for reducing the generation and selection of resistant viruses during treatment, ensuring the binding and neutralization of the mAbs to new variants [18,67,68].

The effectiveness of mAbs depends on the integrity of the recognized viral epitope. Therefore, identifying and cloning Nabs that can specifically target the viral RBD to block viral entry into host cells is a very attractive approach for preventing and treating COVID-19. In this regard, the first step is to seek, identify, and clone effective neutralizing antibodies from the memory B-cell repertoire of recently-infected COVID-19 convalescent patients [19,69,70,71,72]. Fully humanized neutralizing antibodies derived from the memory B cells of recovered patients have higher safety and stability than those derived from immune hybridoma technology and natural phage antibody library technology [73].

The main primarily available mAbs targeting S-RBD with FDA approval and current therapeutic use for the treatment of COVID-19 include casirivimab (REGN10933), imdevimab (REGN10987), bamlanivimab (LY-CoV555), etesivimab (LY-CoV016), tixagevimab (AZD8895), cilgavimab (AZD1061) and, in randomized clinical trials, sotrovimab (VIR-7831/LY-CoV016), MW33, XAV-19, regdanvimab (CT-P59), amubarvimab (BRII-196), and amubarvimab (BRII-198). An overview of clinical trials testing the prophylactic and therapeutic effectiveness of anti-Spike mAbs against COVID-19 is provided in Table 1.

Dumet et al. carried out an in silico analysis and described the possible mAbs combinations that might represent new therapeutic opportunities, discarding those that may be ineffective due to the possible overlapping of epitopes. An example of mAbs that do not overlap the same epitope and are currently being tested as a cocktail in a phase 3 clinical trial is tixagevimab + cilgavimab, while tevesimab + bamlanivimab and casirivimab + imdevimab have already been authorized [83].

### 4.2. Spike Neutralizing nAbs: Casirivimab and Imdevimab

Casirivimab and imdevimab are two recombinant human neutralizing antibodies belonging to the IgG1 class, which bind to the RBD region and, thus, block the attachment of SARS-CoV-2 to the human ACE2 receptor, preventing viral binding to the host cell receptor and its consequent internalization and replication. Their prophylactic and therapeutic use is exclusively recommended in a cocktail (discarding an overlap or mutual competition between nAbs). Their simultaneous use can enhance the neutralizing effect by blocking viral spread. This combination has also been theorized to limit the development of viral mutations [84,85]. The effectiveness of their use as a cocktail has been successfully tested in vitro against the variants B.1.1.7 (Alpha/U.K. origin) [86,87], B.1.351 (Beta/South Africa origin), P.1 (Gamma/Brazil origin), B.1.617.2 (Delta/India origin), AY.1/AY.2 (Delta/India origin), B.1.427/B.1.429 (Epsilon/California origin) [86], B.1.526 (Iota/New York origin) [86,88], B.1.617.1/B.1.617.3 (Kappa/India origin), and C.37 (Lambda/Peru origin). Analysis of the neutralizing activity of casirivimab and imdevimab has shown that they strongly neutralize viruses with the D614G mutation (present in most variants of interest and concern), with an IC50 of 1.69 ng/mL, as well as the variants B.1.1.7 (IC50 of 2.18 ng/mL) and B.1.351 (IC50 of 15.45 ng/mL) [89]. In another in vitro neutralizing assay, Zhou et al. effectively determined the minimal concentration of casirivimab and imdevimab required to neutralize some variants of concern (VOCs). The IC50 values reported for specific VOCs or mutations were: D614G 4.7 ng/mL, B.1.526 (E484K) 6.2 ng/mL, B.1.526 (S477N) 3.9 ng/mL, and E484K 9.2 ng/mL [90].

The authorized FDA dosage is a combination of casirivimab 1200 mg + imdevimab 1200 mg, administered as a single intravenous (IV) infusion over at least 60 min. This dose can be applied from 12 years of age and 40 kg of weight [85]. Analysis of the two different dosages in the antibody cocktail REGN-COV (casirivimab + imdevimab) was tested in a double-blinded randomized trial (NCT04425629) involving 275 outpatient COVID-19 patients. In the treatments, one group received a dose of 2.4 g (1200 mg casirivimab + 1200 mg imdevimab), while another group received a dose of 8 g (4000 mg casirivimab + 4000 mg imdevimab). In clinical or virological parameters, no significant difference between doses was detected. The safety profile was similar between different treatments and the placebo group [54].

In a Phase 3, randomized, double-blind, placebo-controlled study (NCT04452318) with 753 participants, the efficacy and safety of REGEN-COV for preventing COVID-19 infection in household contacts of individuals infected with SARS-CoV-2 was assessed. The primary efficacy endpoint was the development of symptomatic SARS-CoV-2 infection through day 28 in participants who did not have infection or previous immunity (seronegative). In the REGEN-COV group, 11 of 753 participants developed a symptomatic infection (1.5%), while infection was observed in 59 of 752 participants in the placebo group (7.8%), with a relative risk reduction of 81.4% (*p* < 0.001). REGEN-COV treatment also reduced the duration of symptomatic disease (from 3.2 weeks in the placebo group to 1.2 weeks in the treatment group) and the duration of a high viral load (from 1.3 weeks in the placebo to 0.4 weeks in the treatment group). No toxic or adverse effects were detected [77]. Casirivimab/imdevimab treatment may reduce hospital admissions or death (2.4 g: RR 0.43, 95% CI 0.08–2.19; 8.0 g: RR 0.21, 95% CI 0.02–1.79). The relative risk reported for adverse effects of each kind of dose was as follows: 2.4 g, RR 0.76, 95% CI 0.17–3.37; 8.0 g, RR 0.50, 95% CI 0.09–2.73 [91].

### 4.3. Sotrovimab

Sotrovimab is a human recombinant mAb that neutralizes SARS-CoV-1/2 and multiple other sarbecoviruses, targeting a highly conserved epitope that is functionally retained among SARS-CoV-2 variants having a higher barrier to resistance, in comparison with other NAbs that target RBD epitopes with a higher mutation rate. In vitro assays have indicated its effective neutralizing activity against variants of interest and concern, including the alpha, beta, gamma, delta, lambda, and omicron (BA.1 and BA.2) variants [92,93,94,95,96].

These potent effector functions displayed in in vitro assays may result in immune-mediated viral clearance. Sotrovimab contains a two amino-acid Fc modification (termed LS) to increase the half-life and potentially improve bioavailability in the respiratory mucosa through enhanced engagement with the neonatal Fc receptor. This modification may permit therapeutic concentrations for longer durations. In a phase 3, multi-center, randomized, double-blind, placebo-controlled trial (NCT04545060), a single intravenous sotrovimab infusion of 500 mg was evaluated in 430 patients treated, with 438 patients in the placebo group, including a follow-up at 56 days after treatment. In the efficacy outcomes, 1% of the sotrovimab-treated group and 7% in the placebo group had disease progression leading to hospitalization for any cause or death (relative risk reduction of 85% and 97.24%, respectively; CI, 44–96; *p* = 0.002). Serious adverse effects occurred in 2% of the sotrovimab-treated group, compared with the 6% of those who received the placebo. None of the hospitalized patients who received sotrovimab were admitted to the ICU, compared to five patients who received a placebo. These findings suggest that sotrovimab prevents more severe complications of COVID-19, in addition to preventing hospitalization [56,92,93,94,95].

### 4.4. Regdanvimab

Regdanvimab (CT-P59) is a recombinant human mAb G1 which is targeted against the RBD of the Spike protein, thus neutralizing binding between SARS-CoV-2 and ACE2. Regdanvimab potently neutralizes SARS-CoV-2, including the D614G variant, without an ADE effect. The complex crystal structure of CT-P59 Fab/RBD indicates that CT-P59 blocks the interaction regions of RBD for ACE2 receptors with an orientation that is notably different from previously reported RBD-targeting mAbs. The therapeutic effects of CT-P59 have been evaluated in three different animal models (ferret, hamster, and rhesus monkey), demonstrating a substantial reduction in viral titer and alleviation of clinical symptoms. Therefore, CT-P59 may be a promising therapeutic candidate against COVID-19 [97]. Clinical trials (NCT04525079 and NCT04593641) have exhibited a good safety profile in healthy individuals and patients with mild SARS-CoV-2 infection [59,63,85].

In a randomized, placebo-controlled, double-blinded trial phase 2/3 (NCT04602000), treatment with either dose (40 or 80 mg/kg) of CT-P59 decreased hospital admissions or death compared with placebo (RR 0.45, 95% CI 0.14–1.42; RR 0.56, 95% CI 0.19–1.60; 206 participants), but may have increased grade 3–4 adverse events (RR 2.62, 95% CI 0.52–13.12; RR 2.00, 95% CI 0.37–10.70) [98].

### 4.5. MW33

MW33 is a fully humanized IgG1κ SARS-CoV-2 RBD (S1)-targeting mAb with a high neutralization activity, which disrupts the interaction between RBD and ACE2. MW33 was obtained from the blood cells of convalescent patients after B cell screening and single-cell sequencing techniques. FcγRIIB was confirmed to be involved in the ADE of SARS-CoV-2 infection mediated by MW33; nevertheless, introduction of the hIgG1-P329G mutation into the Fc region (MW33/LALA) completely deleted the ADE activity. Potent prophylactic effects against SARS-CoV-2 were observed in rhesus monkeys. Furthermore, an assessment of therapeutic effectiveness indicated viral clearance within just three days. A randomized, double-blind, placebo-controlled, single-dose escalation phase I trial (NCT04533048) demonstrated its favorable safety and pharmacokinetic characteristics. Most of the AEs and abnormal laboratory results were mild, without symptomatic manifestations or the need for medical intervention, and had resolved by the follow-up period. In addition, skin allergies, pruritus, and rash were reported in the 60 mg/kg MW33 dose, and rash in a 60 mg/kg MW33 group was reported [74,78]. A multi-center, randomized, double-blind, placebo-controlled phase II clinical trial (NCT04627584) aimed at evaluating the clinical efficacy and safety of MW33 injection in patients with mild or moderate COVID-19, as well as its pharmacokinetic profile and immunogenicity, is still in progress [78].

### 4.6. XAV-19

XAV-19 is a swine glyco-humanized polyclonal IgG antibody against SARS-CoV-2 RBD. Polyclonal antibodies offer advantages over mAbs in terms of covering the different epitopes of the target antigen and mimicking the natural response to the antigen. Conventional polyclonal heterologous antibodies induce natural human xenogeneic antibody responses, leading to immune complexes and a high risk of serum sickness. To avoid these concerns, CMAH/GGTA1 (CMP-N-acetylneuraminic acid hydroxylase and CMP-N-acetylneuraminic acid hydroxylase) double-knockout pigs were used to produce glyco-humanized polyclonal antibodies (GH-pAbs) lacking Neu5Gc and α-Gal epitopes. In a randomized, double-blind, placebo-controlled phase 2 study (NCT04453384) evaluating the safety, optimal dose, and efficacy of XAV-19 in patients with COVID-19 moderate pneumonia, a single intravenous dose of 2 mg/kg of XAV-19 demonstrated high serum concentrations which were predictive of potent durable neutralizing activity with good tolerability. No hypersensitivity or infusion-related reactions were reported during treatment, and there were no treatment discontinuations due to adverse events. The numerical reduction of nasopharyngeal viral load was more significant with XAV-19 than with placebo (median decreases of viral loads from baseline were 0.6 and 2.8 log10 copies/mL at days 8 and 15, respectively, in the placebo group, and 1.35 and 4.05 log10 copies/mL, respectively, in the XAV-19 2 mg/kg group). Furthermore, the clinical outcomes of COVID-19 did not differ between the groups (i.e., XAV-19-treated patients vs. placebo), but the numbers were too small in this study to accurately determine any trend. According to in vitro experiments, XAV-19 maintains neutralization activity against the most predominant SARS-CoV-2 variants from alpha to omicron (B.1.1.529). Thus, XAV-19 may provide a novel effective therapeutic tool to combat COVID-19 VOCs [99,100,101].

### 4.7. BRII-196 and BRII-198

BRII-196 and BRII-198 are two recombinant humans IgG1 mAbs derived from human B cells isolated from COVID-19 convalescent patients, with a mean terminal half-life of approximately 46 to 76 days. BRII-196 and BRII-198 target distinct epitope regions in the S-RBD protein, demonstrating effective neutralizing activity against VOCs B.1.351, B1.1.7, and P1. BRII-196 and BRII-198 are engineered with a triple amino-acid substitution (M252Y/S254T/T256E [YTE]) in the fragment crystallizable (Fc) region, in order to allow for an extended half-life and reduced Fcγ receptor binding, considering the potential risk of Fc-mediated ADE. During phase I clinical trials (NCT04479631 and NCT04479644), their safety, tolerability, and pharmacokinetics were evaluated in 32 individuals (BRII-196 *n* = 12, BRII-198 *n* = 12, and placebo *n* = 8), and administration was observed to be safe and well-tolerated. No deaths, serious adverse effects, or any systemic or local infusion reactions were detected during the study. A BRII-196 and BRII-198 cocktail is currently under research in ACTIV-2—a platform phase 2/3 clinical study (NCT04518410)—for the treatment of COVID-19 in outpatients [18,70,92,102,103,104]. This treatment demonstrated a statistically significant reduction (by 78%) of relative risk (*p* = 0.00001) of hospitalization and death, compared with placebo, in 837 non-hospitalized COVID-19 patients at high risk of clinical progression [18,105,106].

### 4.8. Criteria to Consider for mAbs Therapy

The use of mAbs is considered one of the most promising approaches for the treatment of COVID-19, due to their high specificity, precise action, long half-lives, low doses required [107], efficacy ranging between 70% to 85% in preventing mortality, and high safety [54,56,58]. They may also be administrated in cocktails to target different epitopes, thus reducing the generation of resistant viruses [60]. The main disadvantage is that their high specificity could be impaired with the appearance of emerging new variants that modify the recognized epitope [68]. Furthermore, mAbs have limited efficacy once the infection is severe. The main risks associated with this type of therapy include allergic reactions [108], acute anaphylaxis, serum sickness, and the generation of antibodies [109]. The most frequent adverse effects are nausea, diarrhea, dizziness, headache, and vomiting, with just 1% reporting grade 2 or higher infusion-related reaction within the first 4 days [110,111]. In vitro data indicate that mAbs targeting SARS-CoV-2 are not associated with ADE [112]. In cases of kidney dysfunction, mAbs have been approved by the Food and Drug Administration (FDA); however, dosage adjustments are required, according to the renal function [113]. During pregnancy or lactation, mAbs appear to reduce the risk of severe disease and no significant adverse maternal or perinatal outcomes have been noted [114,115,116]. Contraindications for certain mAbs have been described for allergic, neoplastic, or infective diseases, as well as hypertension and cardiac failure; however, more studies are required to define their role and establish their possible adverse effects. Furthermore, little is known about their long-term effects (e.g., possible derangement of the immune response and/or increase in neoplasms) [117].

## 5. Immune Response Regulators

A promising pharmacological strategy used for the treatment of severe COVID-19 is the use of molecules whose therapeutic target is regulation of the immune response. These kinds of drugs are mainly focused on the regulation and inhibition of cytokine storm, which is one of the main factors related to the severity of the SARS-CoV-2 infection, leading to rapid disease progression and the consequent high mortality [118]. Blocking the cytokine storm and the hyperinflammatory state is crucial for reducing the COVID-19 death rate.

### 5.1. mAbs Targeting IL-6 and IL-1 Receptor

Severe COVID-19 cases are associated with cytokine storm, which causes organ failure and can lead to death. During a cytokine storm, IL-6 levels are significantly increased in the serum of COVID-19 patients with severe disease. IL-6 is one of the major critical cytokines, which can create an inflammatory storm through activation of the classic signaling pathway (IL-6-mIL-6R-JAK-STAT), the trans-signaling pathway (IL-6-s-IL-6R-JAK-STAT), or an alternative pathway (IL-6-mIL-6R-MAPK/NF-κB). Therapy with anti-mIL-6R/sIL-6R mAbs (e.g., tocilizumab and sarilumab) may help to suppress the cytokine storm in severe cases of COVID-19 [118,119]; however, the consistent message of related studies is that there is no broad-based benefit of IL-6 blockade in COVID-19 patients. Nevertheless, in two of the larger, more extensive trials, there was a clinical benefit in 15–20% of patients when IL-6 blockade was administered early after hospitalization and used in combination with dexamethasone. Thus, the efficacy of IL-6 targeting depends on the underlying health status of the patient, the severity of the disease, and the timing of intervention [120,121,122].

### 5.2. Tocilizumab

One of the earliest therapies for abrogating a cytokine storm was the anti-interleukin-6 receptor mAb tocilizumab (Actemra), developed for the treatment of idiopathic multi-centric Castleman’s disease in the 1990s [79]. Tocilizumab is a genetically engineered mAb, humanized (to decrease antigenicity in the human body) from a mouse antihuman IL-6 receptor antibody using the CDR grafting method, which can recognize both the membrane-bound and the soluble form of IL-6R through competitive blockade of IL-6 binding, thus inhibiting IL-6-mediated signaling and the subsequent inflammatory response. Despite being an IgG1 antibody, a regular dose of tocilizumab in humans causes no antibody-dependent cellular cytotoxicity or complement-dependent cytotoxicity in cells that express IL-6R. Tocilizumab is expected to ameliorate hyperinflammatory diseases in which the over-production of IL-6 is a determining factor. Tocilizumab has been clinically developed as a therapeutic agent for rheumatoid arthritis (RA) and other autoimmune diseases [58,123].

COVID-19 is associated with immune dysregulation and hyperinflammation, including elevated IL-6 levels. The therapeutic effectiveness of tocilizumab in 243 hospitalized patients with severe COVID-19 pneumonia was assessed in a randomized, double-blind, placebo-controlled, phase 3 multi-center study (NCT04320615). Patients treated with tocilizumab received a daily dose of 8 mg/kg of body weight administered intravenously, not exceeding 800 mg. At day 28, 10.6% in the tocilizumab group and 12% in the placebo group had been intubated or died. The findings from this trial indicated that this intervention had no significant effect on the risk of intubation or death, disease worsening, time to discontinuation of supplemental oxygen, or efficacy outcomes. The data did not support the concept that early IL-6R blockade is an effective treatment strategy in moderately ill patients [124].

In another randomized trial (NCT04381936), intravenous tocilizumab doses of 400–800 mg were evaluated in 4116 hospitalized COVID-19 patients. A second dose could be given 12–24 h later, if the patient’s condition had not improved. Furthermore, 82% of patients were receiving systemic corticosteroids at randomization. At day 28, the mortality rate was 29% in the tocilizumab group and 33% in the conventional treatment group. Tocilizumab patients were less likely to reach invasive mechanical ventilation or death (RR 0.85; 95% CI 0.78–0.93; *p* = 0.0005). In hospitalized COVID-19 patients with hypoxia and systemic inflammation, tocilizumab improved survival and other clinical outcomes, adding to the benefits of systemic corticosteroids [125].

In a multi-centered retrospective and observational cohort study in Wuhan, China, 65 COVID-19 patients receiving tocilizumab and 130 not receiving tocilizumab were evaluated. After tocilizumab administration, abnormally elevated IL-6, CRP, fibrinogen, and activated partial thromboplastin time decreased. The detected risk for in-hospital death was lower in the tocilizumab group vs. the non-tocilizumab group (hazard ratio = 0.47; 95% CI = 0.25–0.90; *p* = 0.023). Furthermore, the use of tocilizumab was associated with a lower risk of acute respiratory distress syndrome (OR = 0.23; 95% CI = 0.11–0.45; *p* < 0.0001). Therefore, tocilizumab could be a promising treatment option for prolonging survival in severe COVID-19 patients, by reducing or ameliorating the induced cytokine release syndrome [126].

A previous analysis of 10 randomized clinical trials focused on treating COVID-19 patients with tocilizumab indicated an apparent association between this mAb and improvement in mortality, when compared with placebo (24.4% vs. 29.0%; OR 0.87 [0.74–1.01]; *p* = 0.07; I2 = 10%). Meta-regression suggested a relationship between treatment effect and mortality risk, with benefits at higher levels of risk. Tocilizumab did reduce the need for mechanical ventilation, but did not reduce ICU admission. The evidence thus indicates a short-term mortality benefit, but further research is required [90].

## 6. mAbs Directed at Inflammatory Targets

TNF-α is elevated in acute inflammatory states [127]. Several studies have observed associations between increasing TNF-α and the severity of COVID-19. As it plays a role in amplifying inflammation, it may be a promising therapeutic target. In this line, TNF-α blocking can provide a favorable therapeutic intervention for the treatment of COVID-19.

### Infliximab

Infliximab is a chimeric anti-TNF-α mAb that binds to both soluble and trans-membrane forms of TNF-α at picomolar concentrations. Infliximab has been effectively used for treating RA, reducing serum levels of inflammatory mediators and vascular endothelial growth factors, decreasing the expression of chemokines in the synovial tissue, and reducing lymphocyte migration into the joints of patients with RA [128]. In a phase 2 randomized clinical trial (NCT04425538) assessing the efficacy of a single dose of 5 mg/kg infliximab in 18 hospitalized adult patients with severe or critical COVID-19 (treated previously with remdesivir and dexamethasone), the results revealed an immediate abrogation of inflammatory markers (i.e., significant declines in IFN-γ, TNF-α, IL-6, IL-27, CRP, and ferritin observed explicitly at day three post-treatment), as well as remarkable clinical recovery. Consistent with the pathophysiological role of TNF-α, infliximab rapidly abrogates pathological inflammatory signaling and facilitates clinical recovery in both severe and critical COVID-19. These results suggest that TNF-α blockade may represent a therapeutic strategy offering more precise and durable control of the hyperinflammatory cytokine signature of COVID-19, compared to the broad-spectrum anti-inflammatory effects of steroid therapy [129].

## 7. Drugs That Target Immunomodulatory Pathways

A promising pharmacological strategy used in the treatment of severe COVID-19 is the use of molecules whose therapeutic target is to regulate the immune response. These drugs mainly focus on regulating and inhibiting the cytokine storm and cytokine release syndrome—two processes that can compromise patient survival during SARS-CoV-2 infection [118]. Therefore, blocking the cytokine storm and the hyperinflammatory state are crucial for reducing the death rate associated with COVID-19.

### 7.1. Baricitinib

One of the main drugs that blocks the proinflammatory signaling mediated by cytokines is baricitinib (LY3009104), which has been used successfully in severe and critical COVID-19 cases. Baricitinib intracellularly inhibits the proinflammatory signal of several cytokines (more than 40) by suppressing Janus kinase (JAK) activity, including JAK1/JAK2 and (partially) JAK3, thus blocking STAT activation and inhibiting the expression of related genes. Blocking the JAK–STAT signaling pathway in immune cells is crucial for stopping the excessive and uncontrolled release of cytokines in the cytokine storm context. Baricitinib not only blocks the JAK–STAT signaling pathway but also has high binding affinity to AP2-associated protein kinase-1 (AAK1) and G-associated kinase (GAK), which are two pivotal regulators mediating clathrin-dependent endocytosis, thus reducing viral entry into the cell [20,21,130,131].

A prospective, observational cohort study (NCT04438629) in 20 hospitalized patients with COVID-19 pneumonia, treated with 4 mg baricitinib twice daily for two days followed by 4 mg per day for the remaining seven days, showed a marked reduction in serum levels of IL-6, IL-1β, and TNF-α; there was rapid recovery of circulating T and B cell frequencies, and increased antibody production against the SARS-CoV-2 Spike protein. Furthermore, a clinical reduction in the need for oxygen therapy and a progressive increase in the P/F (PaO2, oxygen partial pressure/FiO2, fraction of inspired oxygen) ratio was observed, in comparison with 56 control patients receiving standard care of hydroxychloroquine or antiviral therapy (lopinavir/ritonavir), either as single agents or in combination. Among the baricitinib-treated patients, 1 of 20 (5%) died after completion of the treatment regimen, compared with 25 (45%) of 56 patients in the non-baricitinib-treated group (*p* < 0.001). No significant difference in ARDS incidence or disease duration between treatments was found. According to this study, baricitinib prevented the progression to a more severe disease by modulating the patient’s immune response and was associated with a more favorable clinical outcome [132].

In another randomized, double-blinded, placebo-controlled clinical trial (NCT04401579) assessing the efficacy of baricitinib plus remdesivir in 515 hospitalized COVID-19 patients, patients receiving baricitinib had a median time to recovery of 7 days, compared with 8 days in the control group, as well as 30% higher odds of improvement in clinical status at day 15 (OR, 1.3; 95% CI, 1.0–1.6). Patients receiving high-flow oxygen or non-invasive ventilation had a time to recovery of 10 days in the baricitinib group, compared with 18 days in the control group. The 28-day mortality was 5.1% in the baricitinib group and 7.8% in the control group (hazard ratio for death, 0.65; 95% CI, 0.39–1.09). In conclusion, the combined therapy of baricitinib plus remdesivir was superior in reducing recovery time and improving clinical status and mortality, mainly among patients receiving high-flow oxygen or non-invasive ventilation. Concerns about the prolonged use of JAK inhibitors—such as immunosuppression, secondary infections, and thrombosis—were discarded, as baricitinib was not associated with a significantly higher incidence of adverse or thromboembolic events. In fact, patients receiving baricitinib plus remdesivir had a significantly lower incidence of adverse events [133].

Some reports have indicated that using JAK inhibitors can interfere with the differentiation of B cells to plasmablasts in a dose-dependent manner, although no alteration was observed in IgG titers [134]. JAK inhibitors can have various impacts on B cells, such as: suppression of activation, differentiation, and class switching; alterations in plasmablast differentiation and immunoglobulin secretion; inhibition of the production of cytokines relevant for activation and survival; down-regulation of antigen-presenting cell function; reduction of responses to CD4+ Th cells, caused by inhibition of the signal transducer and activator of transcription (STAT) phosphorylation [135]. Inhibition of JAK and its influence on cytokine modulation leads to alterations in chemokine production, which can affect B cell trafficking, thus resulting in increased numbers of peripheral B cells. Interferon I/II (IFN-I/II) can compensate for B cell differentiation by inhibiting the cytokine production caused by baricitinib [136]. Further research is required regarding the effects of JAK inhibition on the phenotype of various lymphocyte populations and the quantity and quality of secreted antibodies.

### 7.2. Tofacitinib

Tofacitinib is an orally administered JAK 1/3 inhibitor approved for the treatment of moderate to severe RA which, in the same way as baricitinib, blocks the signaling pathways used by different cytokine receptors. As a consequence, no cellular response is triggered and cytokine production is indirectly suppressed. Decreasing the release of cytokines by Th1, Th17, and many other innate and adaptive immune cells implicated in the pathogenesis of COVID-19 and the action of tofacitinib on multiple critical pathways of the inflammatory cascade may ameliorate progressive, inflammation-driven lung injury in hospitalized patients [137,138,139,140,141].

The efficacy of tofacitinib treatment (10 mg daily up to 14 days) plus standard care in hospitalized participants with COVID-19 pneumonia was assessed in a randomized, multi-center, double-blind, placebo-controlled phase 3 trial (NCT04469114). The incidence of death to day 28 was 18.1% in the tofacitinib group and 29% in the placebo group. Serious adverse events occurred in 14.1% of the tofacitinib group and 12% in the placebo group. Tofacitinib led to a lower risk of death or respiratory failure through day 28, compared with the placebo [137].

In a recent study assessing the effectiveness of tofacitinib therapy in treating severe COVID-19, 30 participants received tofacitinib, while 30 others received standard care. Mortality and the incidence of admission to the intensive care unit were lower in the tofacitinib group than in the control group (16.6% vs. 40.0%, *p* = 0.009; and 15.6% vs. 50.0%, *p* = 0.004). The most remarkable results in the tofacitinib group were a significant decrease in the volume of the affected part of the lungs and a significant increase in oxygen saturation 7 to 10 days after beginning the treatment. Furthermore, the number of patients requiring mechanical ventilation was 0% in the tofacitinib group and 26.7% in the control group. Thus, tofacitinib was effective in managing the cytokine release syndrome in COVID-19. To confirm these findings, other trials with and without the simultaneous use of glucocorticoids are required [142].

### 7.3. Ruxolitinib

Ruxolitinib (INCB018424/INC424) is a JAK 1/2 inhibitor which has been approved by the FDA and EMA for treating polycythemia vera and myelofibrosis, with mild to moderate anemia events being the most common adverse event [143,144]. Ruxolitinib may be effective against elevated levels of cytokines in patients with COVID-19, and it has been hypothesized that the use of JAK inhibitors may affect viral clearance by blocking IFN signaling and the production of SARS-CoV-2 specific nAbs; however, this needs to be further elucidated [145]. The efficacy and safety of ruxolitinib in treating severe COVID-19 have been assessed in a phase II randomized trial in hospitalized patients. In the treatment group, 43 patients received ruxolitinib plus standard care, while 21 received a placebo based on standard care. A remarkable result was that 90% of patients in the ruxolitinib group showed a computed tomography improvement at day 14, compared with 61.9% of patients in the control group (*p* = 0.0495). The mortality at day 28 in the control group was 14.3%, compared with no deaths in the ruxolitinib group. Furthermore, the levels of IL-6, Nerve growth factor-beta (NGF-β), IL-12 (p40), macrophage migration inhibitory factor (MIF), macrophage inflammatory protein 1α (MIP-1α), MIP-1β, and vascular endothelial growth factor (VEGF) were significantly decreased only in the ruxolitinib group. Ruxolitinib recipients had a numerically faster clinical improvement [145].

In another phase II study (NCT04414098) assessing the efficacy of ruxolitinib (5 mg/12 h) in reducing the proportion of patients with COVID-19 who become critically ill (measured by the requirement of mechanical ventilation and/or FiO2 ≥ 50%), the results did not show a significant reduction in COVID-19 pneumonia patients requiring ICU admission and mechanical ventilation; however, a trend indicating a lower mortality rate in critically ill patients receiving ruxolitinib was observed. At the end of the follow-up, 100% survival was observed in the ruxolitinib group, compared with 95% in the control group [146]. Increased survival under ruxolitinib treatment in COVID-19 patients requiring mechanical ventilation is consistent in various studies. In a phase II trial (NCT04359290), 100% of 13 critically ill patients requiring mechanical ventilation survived for at least 28 days during treatment with ruxolitinib. These data suggest that ruxolitinib might be efficacious in COVID-19-induced ARDS patients requiring invasive mechanical ventilation [147].

### 7.4. Statins

Statins are HMG-CoA (3-hydroxy-3methylgultaryl-coenzyme A) reductase inhibitors which are used widely to treat hypercholesterolemia. They can reduce LDL levels more than other cholesterol-lowering drugs, and lead to lower triglyceride levels in hypertriglyceridemic patients. They are well-tolerated and have an excellent safety record [148].

Statins are known for their pleiotropic anti-inflammatory effects, including augmentation of ACE2 expression and inhibition of the Toll-like receptor (TLR)-MYD88-NF-kB pathway [149]. Statins also reduce C-reactive protein in patients with cardiovascular disease; therefore, they may have potential anti-inflammatory benefits in COVID-19 patients [150]. However, in a previous study on SARS-CoV infections (NCT00979121), statins failed to confer any benefit in ARDS, as measured by survival. This study was conducted in 745 patients who received rosuvastatin or placebo, and no significant differences between the rosuvastatin and placebo groups regarding hospital mortality (primary outcome, 29% vs. 25%, *p* = 0.21) or ventilator-free days (15 ± 11 vs. 15 ± 11, respectively; *p* = 0.96) was observed [151,152]. Moreover, the adverse outcomes from randomized controlled trials of statin treatment in mechanically ventilated ARDS and sepsis patients have contributed to a reluctance to consider statins as a complementary treatment in patients with COVID-19 infection [152].

However, statins could mitigate the effects of COVID-19 in selected patients, based on specific criteria such as associated coagulopathies, endothelial dysfunction, and unregulated inflammation. Therefore, further evidence from clinical trials is required to confirm this hypothesis and detail the dose and type of statin administered [150].

One study (NCT04407273) assessed the effect of statin therapy on hospital mortality in 2157 COVID-19 patients: 581 patients were treated with statins plus conventional treatment, while the rest were treated with conventional therapy (control). During the study, 353 deaths occurred. There was a significantly lower mortality rate in statin-treated patients than in the non-statin group (19.8% vs. 25.4%; chi2 with Yates continuity correction: *p* = 0.027). In patients who maintained statin treatment throughout their hospitalization stay (*n* = 336), the mortality was even lower (17.4%; *p* = 0.045) than that in the non-statin group. The Cox model applied to the cause-specific hazard function (HR = 0.58; C.I. 0.39–0.89; *p* = 0.01) and the competing-risks FG model (HR = 0.60; CI = 0.39–0.92; *p* = 0.02) suggested that statins are associated with reduced COVID-19-related mortality. According to the contrasting results of the latest clinical trials, statin therapy should not be discontinued until broader clinical trial results are obtained [153].

### 7.5. Anakinra

Anakinra is an anti-interleukin-1 receptor (IL-1R) antagonist, which was approved for the treatment of RA more than 12 years ago [154]. Anakinra has been shown to offer significant clinical benefits in patients with COVID-19 and a systemic hyperinflammation state. A systematic review and meta-analysis regarding the impact of anakinra on the outcomes of hospitalized patients with COVID-19, in which six studies involving patients with moderate to severe pneumonia (*n* = 1553) were evaluated, yielded a hazard ratio for death in patients treated with anakinra of 0.47 (HR 0.47; CI = 0.34–0.65). A multivariate meta-regression analysis did not reveal any significant associations between the mean age, percentage of males, and mean administration time since onset of symptoms among the included studies, as well as the hazard ratios for death (i.e., 28-day mortality). Furthermore, there was no association between the daily dose of anakinra during the first three days of administration and the hazard ratios. The main result of this meta-analysis was a 50% decrease in the adjusted risk of death in hospitalized patients with moderate-to-severe COVID-19 in patients treated with anakinra, compared with patients that did not receive anakinra. Early administration tended to have a more significant beneficial effect than late administration. As clinical trials evaluating the effectiveness of anakinra in COVID-19 remain scarce, and the preliminary results from observational studies are beneficial, the application of this treatment should be considered in future studies [155].

### 7.6. Fluvoxamine

Fluvoxamine is a selective serotonin reuptake inhibitor (SSRI), used widely since the 1990s, mainly in the therapy of obsessive–compulsive disorder [156]. Fluvoxamine has been consistently considered the most potent S1R agonist clinically available, which may effectively reduce cytokine production and prevent clinical deterioration. Among its main benefits, fluvoxamine is inexpensive, easy to use, widely available globally, and highly lipophilic, with rapid intracellular uptake into lung epithelial cells [157,158].

In a randomized, double-blind clinical trial (NCT04342663) assessing the effectiveness of fluvoxamine as a potential treatment to reduce clinical deterioration and mortality in non-hospitalized individuals with confirmed COVID-19, 80 participants were treated with 100 mg of fluvoxamine daily for 15 days, compared with placebo (*n* = 72). No clinical deterioration occurred in the fluvoxamine-treated group, compared with six patients in the placebo group (absolute difference 8.7%; 95% CI, 1.8–16.4%; log-rank χ^2^ = 6.8; *p* = 0.009). At 14 days, no patients receiving fluvoxamine were hospitalized for clinical deterioration; however, 12.5% of patients who declined treatment were hospitalized (*p* = 0.005). During this study, no patients died. To determine clinical efficacy, larger and more extensive randomized clinical trials are necessary [159].

In a recent clinical trial (NCT04727424) assessing the efficacy of fluvoxamine (100 mg twice daily for 10 ten days) vs. placebo (conventional treatment) in preventing hospitalization and death in acutely symptomatic patients, 741 patients were allocated to fluvoxamine and 756 to placebo. From the results, the number of hospitalized patients was lower in the fluvoxamine group than in the placebo group (11% vs. 16%; RR 0.68, 95% C.I. 0.52–0.88). Furthermore, there were 17 deaths in the fluvoxamine group and 25 in the placebo (OR 0.09; 95% C.I. 0.01–0.047). No significant adverse effects were reported between treatments. In conclusion, fluvoxamine treatment administered in early diagnosed COVID-19 reduced the risk of hospitalization. Recent evidence from clinical trials has revealed a potential therapeutic role of fluvoxamine in COVID-19 patients if it is administered in an early stage (i.e., just after detection) and at a correct dose (around 100 mg twice daily for 10 days).

Other studies (NCT04718480, NCT05087381, NCT04885530, NCT04510194) are currently being carried out to determine the efficacy of fluvoxamine in the treatment of COVID-19, from which critical information will be collected to evaluate the effect of its implementation and to determine the correct time at which it must be administered after diagnosis [160].

### 7.7. PI3K/Akt/mTOR Pathway Inhibitors

COVID-19 disease progression is associated with anti-viral T cell exhaustion—typically characterized by high programmed cell death protein 1 (PD-1) expression—which occurs in the early stages of infection [161]. Dysregulation of T-cell functions precedes the cytokine storm (uncontrolled immune response) and neutrophil expansion in alveolar tissues, leading to tissue damage [162]. Hyperinflammation (recognized by fever with elevated C-reactive protein) and increased coagulation (recognized by an increase in D-dimer and prothrombin time with reduced fibrinogen) [163], as well as T cell function and cytokine production, can be manipulated by targeting the phosphoinositide 3-kinase/protein kinase B/Mammalian target of rapamycin (PI3K/Akt/mTOR) pathway [164]. Additionally, the PI3K-δ isoform is thought to be the main functional regulator of CD8 [165], Tregs [166,167], B cells, mast cells [168], and neutrophils [169]. Furthermore, mutations in PI3K-δ have been associated with increased risk of respiratory infection, suggesting it as a potential therapeutic target [170,171]. Some drugs that target different components of the PI3K/Akt/mTOR pathways have already been approved, and their potential to manage COVID-19 could be explored.

The FDA-approved inhibitors for the PI3K/Akt/mTOR pathway include: Idelalisib, which targets PI3K-δ and is prescribed for some lymphomas; Copanlisib, which targets Pan PI3K and is prescribed for elapsed follicular lymphoma; Duvelisib, which targets PI3K-δ and PI3K-γ and is prescribed for follicular and lymphocytic lymphoma; Al-pelisib, which targets PI3K-α and is prescribed for some types of cancer; Umbralisib, which targets PI3K-δ and is prescribed for marginal zone and follicular lymphoma; Rapamycin, which targets mTOR and is prescribed for lymphangioleiomyomatosis and renal transplant; and Everolimus, which targets mTOR and is prescribed for some types of carcinoma. There are no approved Akt inhibitors at present, but some are being tested in clinical trials [172]. At least six clinical trials involving inhibitors of the PI3K/Akt/mTOR pathway have been carried out (3 completed without publications and 3 on course), including two completed studies for Duvelisib (NCT04372602 and NCT04487886), one completed (NCT04409327) and two active (NCT04948203 and NCT04584710) for Sirolimus, and one active (NCT04444401) for Everolimus. Once the published results are available, a more exact evaluation of the efficacy of such inhibitors in the treatment of COVID-19 may be possible.

### 7.8. Novaferon

Novaferon is a genetically engineered drug derived from 12 sub-types of human interferon, which is ten times more effective than human Interferon-alpha-2b. This drug was approved in 2009 in China as a treatment for chronic hepatitis B due to decreased viral clearance [173,174]. In China, a randomized, open-label, multi-center clinical trial was conducted in three groups of COVID-19 patients. The first group was treated with 40 µg novaferon twice daily by inhalation (30 patients), the second group (30 patients) with novaferon plus lopinavir/ritonavir (200/50 mg twice daily), and the last group only lopinavir/ritonavir (29 patients, two tablets twice daily). The two groups that received novaferon presented an increased percentage of viral clearance at three days (N: 16.7%; N + L/R: 36.7%; L/R: 10.3%), six days (N: 50%; N + L/R: 60%; L/R: 24.1%) and nine days (N: 56.7%; N + L/R: 70%; L/R: 51.7%) [174]. Conducted clinical trials considering novaferon have placed this drug as a promising therapeutic against COVID-19; however, more controlled clinical trials are needed to validate the obtained results.

### 7.9. Vitamin D

Vitamin D is a steroid hormone which is vital for modulating the immune system and maintaining serum calcium homeostasis. It is obtained mainly from sunlight exposure and dietary supplementation [175,176]. Vitamin D is present in two isoforms: D2 (ergocalciferol), which is synthesized from yeast, sunlight exposed-mushrooms, cod liver oil, oily fish, egg yolks, and plants; and vitamin D3 (cholecalciferol), which is synthesized endogenously from 7-dehydrocholesterol in the skin dermis upon exposure to sunlight (UVB radiation at 290–315 nm). Notably, vitamin D3 is more easily absorbed by chylomicrons than vitamin D2 [177,178,179,180].

The clinically accepted serum biomarker for vitamin D status is 25-hydroxyvitamin D [25(OH)D] [181]. Vitamin D has diverse immunomodulatory effects on innate immunity, as demonstrated by two observations in which the receptor for 1,25(OH)2D (vitamin D receptor, VDR) is detectable in activated, proliferating lymphocytes. Furthermore, monocytes/macrophages from patients with the granulomatous disease sarcoidosis constitutively synthesize the active form of vitamin D, 1,25-dihydroxy vitamin D (1,25(OH)2D) from precursor 25-hydroxyvitamin D (25OHD) [181]. Studies have shown that T and B cells express vitamin D receptors (VDRs) after activation, which are mainly up-regulated in the proliferation stages [182]. After activation of the VDR, relevant signaling pathways regulate the expression of a large number of target genes (~1000) in lymphocytes, monocytes, macrophages, and dendritic cells [183,184,185]. T-cells are direct targets for 1,25(OH)2D, which potently modulates the T-cell phenotype, promoting the development of suppressor regulatory T-cells (Tregs) [182]. Therefore, the translocation of T cells can be influenced by 1,25(OH)2D by stimulating T-cell expression of chemokine receptor 10 (CCR10), which recognizes the chemokine CCL27 secreted by epidermal keratinocytes and promotes the translocation or retention of T cells in a given tissue. Results from murine knockout models have suggested that vitamin D is also involved in T-cell homing within the gastrointestinal tract [186,187]. As mentioned above, vitamin D is essential in regulating the immune system and, thus, may affect the response to COVID-19 infection [188,189].

Countries proximal to the equator have been found to exhibit lower levels of COVID-19 fatalities than those further from the equator; furthermore, a lower amount of vitamin D is produced during the winter season, and the reported vitamin D deficiency in COVID-19 fatalities may be UV-related. These data indicated a dependence on latitude and location regarding COVID-19 susceptibility [190,191,192]. A retrospective study of 216 COVID-19 patients from Spain showed a higher frequency of vitamin D deficiency (<20 ng/mL) in patients than in controls (82.2% vs. 47.2%, respectively). Furthermore, 25OHD was inversely correlated with serum ferritin (*p* = 0.013) and D-dimer levels (*p* = 0.027). However, no significant correlation was found between vitamin D deficiency and COVID-19 severity [193]. A meta-analysis assessing data from 20 European countries found a negative correlation between vitamin D levels and COVID-19 morbidity/mortality. Moreover, a significantly lower level of vitamin D (*p* = 0.004) was found in PCR-positive COVID-19 patients, compared with negative COVID-19 patients [194,195].

Based on the above, vitamin D supplementation could be recommended during COVID-19. However, a concern that may arise is the possibility of intoxication. Acute vitamin D toxicity, defined by blood 25(OH)D, is indicated by a concentration greater than 150 ng/mL. Chronic toxicity appears after a daily intake of >40,000–60,000 IU for ≥1 month consecutively. Therefore, the possibility of vitamin D toxicity is slight [196]. A randomized, double-masked clinical trial (NCT04366908) assessing the ability of calcifediol to reduce the need for admission to ICU and related death in 66 COVID-19 hospitalized patients demonstrated that administration of a high dose of Calcifediol or 25-hydroxyvitamin D (60,000 IU of vitamin D3 or placebo daily for 7 days) significantly reduced ICU admission. This supplementation also conferred a significantly greater number of SARS-CoV-2 RNA-negative patients by day 21, and fibrinogen was also significantly decreased in treated patients. Moreover, there was 100% survival in the calcifediol-treated group, compared with 2 fatalities in the placebo group [197].

A meta-analysis has evaluated 10 selected systematic reviews (published until January 2022) assessing the efficacy of vitamin D supplementation in reducing the severity of COVID-19, measured according to the reduction of morbidity and mortality in hospitalized COVID-19 patients. The results provided strong evidence that vitamin D supplementation reduces the risk of mortality (Odds ratio: 0.48, 95% CI: 0.346–0.664; *p* < 0.001), as well as the need for intensive care (Odds ratio: 0.35; 95% CI: 0.28–0.44; *p* < 0.001) and mechanical ventilation (Odds ratio: 0.54; 95% CI: 0.411–0.708; *p* < 0.001). The authors concluded that vitamin D supplementation can act as an adjuvant in COVID-19 therapy, due to its effectiveness in reducing the severity of the disease [198]. Nevertheless, another systematic review and meta-analysis addressing the association of serum vitamin D levels and COVID-19 mortality from 21 studies (2 case-control and 19 cohort studies), published by April 2022, reported that vitamin D deficiency was associated with COVID-19 mortality in the overall analysis, but not when the analysis was adjusted to vitamin D cutoff levels <10 or <12 ng/mL (RR 1.60, 95% CI 0.93–2.27, I2 60.2%), suggesting that confounders may have led to many observational studies incorrectly estimating the association between vitamin D status and mortality. Deficient vitamin D levels were not associated with increased mortality rate in patients with COVID-19 when the analysis included studies with adjustments for confounders. Thus, further randomized clinical trials are required to verify this association [199]. To date, the exact dose of vitamin D for COVID-19 patients to gain any immunomodulatory benefit remains unclear, although the recommended 10 μg daily seems justifiable to maintain serum concentrations of 25-hydroxyvitamin D above 25 nmol/L [200].

### 7.10. Criteria to Consider for Immune Regulator Drug Therapy

The advantages of drugs that can block the JAK–STAT signaling pathway (involved in the cytokine storm) include their broad-spectrum ability to inhibit more than 40 cytokines, in a reversible way, by specifically inhibiting Janus kinase (JAK) activity, as well as their added benefit of reducing viral entry into the cell by blocking clathrin-dependent endocytosis [20,21,142]. However, various adverse effects have been described, such as collateral pneumonia, herpes zoster (main adverse effect at high doses), urinary tract infections, non-melanoma skin cancer, thrombosis, pulmonary embolism, and laboratory abnormalities such as neutropenia, lymphopenia, anemia, thrombocytosis, liver enzyme elevations (ALT, AST), lipid elevations (total cholesterol; low-density lipoprotein, LDL; high-density lipoprotein, HDL), and elevated creatine phosphokinase (CK). Despite the above, JAK–STAT inhibitors are generally well-tolerated [201]. Other risk factors that may lead to venous thromboembolism are older age, obesity, history of DVT/PE, and the use of selective COX-2 inhibitors [202]. Drug adjustments are required in patients with eGFR < 60 mL/min, as 75% of the drugs of this kind are secreted by the kidney. In particular, Baricitinib is not recommended for dialysis patients or those who develop AKI [203].

Although statins are highly effective, safe, and related to reductions in total cholesterol, the risk of a heart attack, and the risk of a stroke, they have also been linked to adverse effects such as muscle pain, digestive problems, mental confusion, the risk of diabetes type 2, and (rarely) liver damage. Some risk factors include being female, old age, kidney or liver disease, hypothyroidism, and amyotrophic lateral sclerosis [204]. Meta-analyses have shown that high doses of anakinra may increase the risk of serious infections, especially when patients have comorbidity factors, and its effectiveness is correlated with its early administration [169]. The side-effects of fluvoxamine are nausea and vomiting (37%), somnolence, dry mouth, and headache (also occurring in over 20%). Approximately 12% of patients could not tolerate fluvoxamine and withdrew from treatment [205]. Vitamin D plays a major role in immune function and exerts anti-inflammatory effects, which may prove to be important in the context of cardiovascular disease (CVD) and COVID-19, as this infection is associated with numerous cardiovascular complications including arrhythmia, myocardial injury, cardio-myopathy, and thrombotic events. To date, studies have indicated the minimal benefit obtained through vitamin D supplementation; however, it remains unknown whether supplementation with vitamin D can mitigate CVD complications derived from COVID-19 [206,207]. Even though vitamin D intoxication is rare, it does occur at doses of 50,000 IU/day (serum levels of 50 ng/mL), leading to hypercalcemia and with the most frequent symptoms being vomiting, dehydration, pain, and loss of appetite [208].

## 8. Viral Polymerase Inhibitors

After the virion releases the viral genome into the host cell cytoplasm, the viral cycle of SARS-CoV-2 is initiated by translation of ORF1a and ORF1b for expression of the polyproteins pp1a and pp1ab, which are cleaved by viral proteases (Mpro, 3CLpro, and PLpro) and host cell proteases for the production of non-structural proteins (NSPs) that form the RNA-dependent RNA polymerase (RdRp) complex that synthesizes sub-genomic RNAs and RNA (+) for the new virion [209,210,211]. Blocking any of these mechanisms can prevent viral replication. Therefore, several strategies are focused on this topic; Table 2 lists some of these having the most significant results.

### 8.1. Remdesivir

Remdesivir (GS-5734) is an antiviral prodrug against Ebola virus, Nipah virus, MERS, and SARS-CoV. Being a nucleotide analog that is phosphorylated in its active form in the host cell, remdesivir competes with cellular ATP for the inhibition of viral RdRp, due to it being positioned as a substrate for nascent viral RNA strands, causing delayed termination of replication due to the free 3′-OH group of the analog [217,218,219]. In China, a multi-center, double-blind, randomized clinical trial has been conducted in 158 participants diagnosed with severe COVID-19, who were treated with remdesivir (200 mg on day 1 and 100 mg after that until day 10), as well as 79 patients treated with a placebo. From the results, remdesivir decreased the length of hospital stay (21 days vs. 23 days) and time on invasive mechanical ventilation (7 days vs. 15.5 days); however, no significant differences were observed in terms of mortality rate and clinical improvement [220].

In a randomized, multi-center, open-label phase 3 clinical trial carried out in patients with severe COVID-19, a comparison was made between 5 and 10 days of remdesivir administration (200 mg on day 1 and 100 mg after that until day 10). No significant difference between 5 or 10 days of treatment was found; nevertheless, 5-day treatment reduced the mortality rate, decreased the time for clinical improvement, and favored non-invasive mechanical ventilation requirements [221]. Furthermore, in a comparison between the clinical trial of Goldman et al. (2020) and a cohort study of 818 patients with severe COVID-19 who received standard care, remdesivir increased the time to clinical improvement at day 14 (74.4% vs. 59.0%) and decreased the mortality rate (7.6% vs. 12.5%) [222]

Contrary to previous evidence, in an open-label, multi-center, randomized, controlled, phase 3 clinical trial (DisCoVeRy) considering hospitalized COVID-19 patients with oxygen requirements, remdesivir (200 mg on day 1 and 100 mg after that until day 9) did not present significant differences from standard care in terms of clinical improvement, requirement for invasive mechanical ventilation, and mortality [223].

In Canada, a randomized controlled clinical trial was conducted in hospitalized COVID-19 patients, of whom 634 received remdesivir (200 mg per day 1 and 100 mg after that until day 10) and 648 patients received standard care. Remdesivir decreased mortality (18.7% vs. 22.6%), the percentage of invasive mechanical ventilation requirement (8% vs. 15%), and the average per day of 28 without oxygen supplementation (15.9 days vs. 21.4 days) [224]. The efficacy results from clinical trials in severe COVID-19 patients affirm why the FDA cleared remdesivir—on 22 October 2020—as the first treatment for emergency use in hospitalized pediatric and adult COVID-19 hospitalized patients, as well as those non-hospitalized patients with a high risk of progressing to severe COVID-19.

### 8.2. Ribavirin

Ribavirin (1-f8-D-Ribofuranosil-1,2,4-triazole-3-carboxamide) is a guanosine analog used to treat respiratory syncytial virus, hepatitis C virus, SARS-CoV, and MERS, with three known antiviral mechanisms. The first is that ribavirin triphosphate is incorporated by viral polymerase into nascent RNAs, causing elongation to stop; the second is by inhibiting the inosine monophosphate dehydrogenase enzyme involved in the synthesis of cellular GTP; and the third mechanism is the increased regulation of genes stimulated by interferon [225,226,227]. A multi-center, open-label, randomized phase 2 clinical trial was conducted in 86 COVID-19 hospitalized patients, who received interferon beta-1b (8 × 10^6^ IU every other day), lopinavir/ritonavir (400/100 mg twice daily), or ribavirin (400 mg twice daily) for 14 days, compared to 42 patients receiving standard care (lopinavir/ritonavir). As a result, the combination therapy decreased viral clearance time (7 days vs. 12 days) and length of hospital stay (9 days vs. 14.5 days) [213].

Elalfy et al. (2021) conducted a clinical trial testing the antiviral combination of nitazoxanide, ribavirin, ivermectin, and zinc compared to standard care, where the combination led to better viral clearance (see the section on nitazoxanide). Although clinical trials on ribavirin frequently consider it in combination with other antivirals, the use of this drug is effective. As such, the combination of drugs could be a limiting factor in discovering their individual effectiveness and, so, more single-use clinical trials are needed [228].

### 8.3. Favipiravir

Favipiravir (6-fluoro-3-hydroxy-2-pyrazine) is an antiviral drug used for treatment of influenza A, B, viral hemorrhagic fever, and Ebola, due to its capacity to inhibit RdRp. Different trials using this drug have been conducted to assess its therapeutic utility for SARS-CoV-2, due to the homology in its viral replication cycle [214,229,230]. A randomized study comparing Favipiravir with Lopinavir/Ritonavir conducted in patients with moderate or severe COVID-19 pneumonia indicated no difference between the two groups in terms of mortality, number of intubated patients, recovery time, and hospital stay [214]. Although favipiravir has been considered a promising drug for severe COVID-19, the results of clinical trials are not promising enough for it to be considered as a primary drug in this context.

### 8.4. Molnupiravir

Molnupiravir (EIDD-2801) is a pyrimidine analog prodrug with antiviral properties that inhibits respiratory syncytial virus, influenza (H1N1), Ebola, Chikungunya, noroviruses, and coronaviruses. Its active metabolite is the ribonucleoside analog β-d-N4-hydroxycytidine (EIDD-1931). This is transformed by esterases to 5′-triphosphate EIDD-1931, which competes as a substrate for RdRp with cytidine triphosphate and uridine triphosphate, inducing multiple mutations in nascent RNA and consequently damaging the virus. In addition, molnupiravir is a highly reducing agent, which can affect the environment required for viral infection [231,232,233]. A randomized, double-blind, Phase 2 clinical trial in patients with mild COVID-19 compared doses of 200, 400, and 800 mg of molnupiravir (twice daily for five days; 23, 62, and 55 patients, respectively) with placebo (62 patients). The group with the best results was the one that received 800 mg of molnupiravir, when compared with the control group, as it decreased the time (800 mg M: 14 days vs. C: 15 days) and the percentage of viral clearance at four weeks (800 mg M: 92.5% vs. C: 80.3%) [234].

Another randomized, double-blind, controlled Phase 3 clinical trial was performed in 716 unvaccinated patients with mild COVID-19 having at least one risk factor for severe COVID-19. They were treated with 800 mg molnupiravir (twice daily for five days) and compared with 717 patients in the control group (placebo). On day 29, molnupiravir decreased hospitalization or mortality (M: 6.8% vs. Control: 9.7%) and adverse events (M: 30.4% vs. Control: 33%) [235]. The clinical trials conducted so far indicate molnupiravir as a promising drug against COVID-19; therefore, on 23 December 2021, the FDA approved it for treatment in adult patients with mild or moderate COVID-19 at high risk of progression to severe COVID-19. However, further clinical trials are required to elucidate its benefits.

### 8.5. Sofosbuvir and Daclastivir

Sofosbuvir and daclastivir are direct-acting antivirals approved for the treatment of hepatitis C virus (HCV) infection. Sofosbuvir is a uridine monophosphate nucleotide (2′ Me-F) which is incorporated during viral replication, causing viral RNA synthesis to stop. It has been observed to inhibit Zika, yellow fever virus, and Chikungunya viral replication. Daclastivir (BMS-790052) is an inhibitor of the HCV NS5A viral protein (formation of the viral replication complex), preventing the normal functioning of the viral polymerase (NS5B RdRp) [236,237,238,239]. An open-label, randomized, controlled, multi-center clinical trial was conducted in Iran, providing patients having moderate or severe COVID-19 with 400/60 mg sofosbuvir/daclastavir for 14 days (33 patients). Compared to standard care (33 patients; hydroxychloroquine and lopinavir/ritonavir), sofosbuvir/daclastavir showed greater clinical improvement at 14-day discharge (S/D 88% vs. 67%), shorter hospital stay (median S/D 6 days vs. 8 days), and a decrease in the number of deaths (S/D 3 deaths vs. 5 deaths) [240]. An open-label clinical trial has been performed in patients with severe COVID-19, where administration of 400/60 mg sofosbuvir/daclastavir once daily for 14 days (35 patients) was compared with administration of 600 mg ribavirin every 12 h for 14 days (27 patients). Sofosbuvir/daclastavir was found to decrease the length of hospital stay (S/D: 5 days vs. R: 9 days) and mortality rate (S/D: 6% vs. R: 33%) [241]. In Egypt, a randomized, prospective, multi-center clinical trial was conducted in patients with pneumonia caused by COVID-19, where a group of 96 patients were administered sofosbuvir/daclastavir (400 mg/60 mg once daily for 14 days). Compared with a control group of 78 patients who received standard care, sofosbuvir/daclastivir decreased the mortality rate (14% vs. 21%), shortened the hospital stay (9% vs. 12%, *p* < 0.01), and increased viral clearance (84% vs. 47%) [242]. The “DISCOVERY” clinical trial conducted in Iran in patients hospitalized by COVID-19 considered the double-blind, placebo-controlled, and randomized administration of 400/60 mg sofosbuvir/daclastavir once daily for 10 days (541 patients). Compared with the group that received placebo plus standard care, no statistical difference was observed in terms of mortality (S/D 11% vs. 10%) or medical discharge at 10 days (S/D 77% vs. 76%) [243]. The initial clinical trials, considering a low number of patients, yielded promising results for sofosbuvir/daclastavir in terms of clinical improvement; however, the more recent clinical trial performed with a considerable sample size differed considerably from these initial clinical trials. Therefore, more studies are required to confirm whether sofosbuvir/daclastavir should be recommended for the treatment of COVID-19.

### 8.6. Criteria to Consider for Viral Polymerase Inhibitors

Viral polymerase inhibitors are considered the most promising drugs against COVID-19, as they have the ability to directly target SARS-CoV-2 replication. Remdesivir was the first drug approved by the FDA for cases at risk of severe COVID-19 progression, supported by clinical trial results indicating decreased ICU requirement, mortality rate, hospital stay, and better clinical improvement. However, the dose of Remdesivir should be tested in people with renal impairment, in order to avoid toxicity, as 50% of the active metabolite is excreted in the urine and its drug vehicle sulfobutylether-β-cyclodextrin (SBECD) can accumulate in the kidney [244]. Furthermore, adverse effects of remdesivir have been reported in pregnant women with critical COVID-19, such as spontaneous fetal loss, induced abortions, and stillbirths [245]. Ribavirin and favipiravir decrease the time to resolution of the disease with a shorter viral clearance time. The first results obtained with sofosbuvir/daclastivir placed it as a drug with positive results in the context of severe COVID-19, as it appeared to decrease hospital stay and mortality rate; however, studies with larger populations have indicated no significant difference between this drug and standard care. There are no contraindications for favipiravir in renal pathologies; however, more than 90% of its excretion is through the urine, so its use is recommended only in patients with a GFRe > 30 mL/min [244,246]. It has also been reported that favipiravir has teratogenic effects in animals and, so, it may have adverse effects in pregnant women and breastfeeding is not recommended if on treatment [247,248]. Sofosbuvir/daclastivir has not been reported to have serious adverse effects (nausea, vomiting, and insomnia); however, it is recommended not to use it in conjunction with drugs that inhibit CYP3A4 and P-gp enzymes—which are the main targets—in order to avoid possible complications [249]. Molnupiravir is a drug that has been used for mild or moderate COVID-19; however, it is a promising drug for preventing progression in patients with mild COVID-19 who present risk factors associated with high probability to progress to severe COVID-19.

In general, antiviral nucleoside analogues are of concern due to their possible mutagenic role in host cells. Molnupiravir is known to be transformed in plasma to its active ingredient β-d-N4-hydroxycytidine (NHC), which presents a risk of off-target mutagenesis; notably, Zhou et al. have reported that ribonucleosidated NHC induces DNA mutagenesis in dividing cells in vitro [235,250,251,252]. Therefore, the use of other antiviral options or monoclonal antibodies is recommended prior to its administration. Favipiravir and rivabirin are drugs with active compounds similar to molnupiravir; however, their mutagenicity in host cells has not been reported, as molnupiravir is 100 times more active against SARS-CoV-2, in comparison [250,251].

## 9. Corticosteroids

Corticosteroids, as suppressors of the acute inflammatory response, are some of the most-used treatment options. Thus, some clinical trials have assessed their use for the treatment for COVID-19 (see Table 3).

### 9.1. Dexamethasone

Dexamethasone (C22H29FO5) is a synthetic glucocorticoid used to inhibit the acute inflammatory response by suppressing chemokine and proinflammatory cytokines [255,256]. In a randomized, open-label, multi-center clinical trial (NCT04327401) in-volving 299 patients with moderate or severe COVID-19, 151 patients were given dexamethasone plus standard care (20 mg intravenous per day for five days and then 10 mg IV per day for five more days or at discharge), while 148 patients received standard care. Patients receiving dexamethasone had significantly increased mechanical ventilation-free days at 28 days (dexamethasone 6.6 days vs. standard care 4 days, *p* = 0.04) and lower mean SOFA (Sequential Organ Failure Assessment) score (dexamethasone 6.1 vs. standard care 7.5, *p* = 0.04) [257].

The clinical trial “RECOVERY” (NCT04381936), an open-label, randomized, con-trolled study, evaluated the administration of dexamethasone in 2104 hospitalized COVID-19 patients (6 mg once daily for ten days, oral or intravenous), compared to a group of 4321 patients who received standard care for 28 days. The patients treated with dexamethasone had a lower mortality rate than the standard care group (22.9% and 25.7%, respectively; *p* < 0.001). In addition, the sub-groups that received invasive mechanical ventilation (dexamethasone 29.3% vs. standard care 41.4%) and those that did not receive oxygen through invasive mechanical ventilation (dexamethasone 23.3% vs. standard care 26.2%) presented a lower incidence of death in the group that received dexamethasone [258].

Subsequently, when observing the efficacy of dexamethasone in preventing mortality in patients with moderate or severe COVID-19, the efficacy and safety of different concentrations of dexamethasone have been evaluated in various clinical trials. In a randomized, blinded, controlled, and multi-center clinical trial carried out in Europe and India called “COVID STEROID 2”, the concentrations of 12 mg (491 patients) and 6 mg (480 patients) of dexamethasone were compared in patients with severe COVID-19 for 10 days. They observed no statistical significance in mortality, serious adverse effects, and the number of days without mechanical ventilation [259].

In India, a three-arm randomized clinical trial (IRCT20100228003449N31) was conducted in patients diagnosed with moderate or severe COVID-19, where one group received 8 mg of intravenous dexamethasone per day (low dose, 47 patients), another group received 8 mg of intravenous dexamethasone twice daily (intermediate dose, 40 patients), and the last group received 8 mg of intravenous dexamethasone three times a day (high dose, 46 patients). No significant differences were observed between the groups; however, the patients who received the low dose had lower mortality and greater clinical recovery, while the intermediate and high doses were associated with more serious adverse effects [260].

In a randomized, multi-center, open-label clinical trial (NCT04395105) in patients with severe COVID-19, 49 patients were administered 16 mg of dexamethasone daily (intravenous for five days, high dose), and 49 patients were administered 8 mg or 6 mg of intravenous dexamethasone daily for 5 or 10 days (low dose), respectively. When comparing both groups at 28 days, no statistical significance was found in mortality, days without invasive mechanical ventilation, and infection rate; however, patients treated with high doses of dexamethasone had fewer days without invasive mechanical ventilation (12 days), compared to the low dose group (19 days) [261]. According to the clinical trials that have been performed, the results do not support the theory that higher concentrations of dexamethasone produce greater clinical improvement, when compared to low doses.

### 9.2. Methylprednisolone

Methylprednisolone is a synthetic glucocorticoid intermediate action that, in pneumonia, positively regulates the resolution of pulmonary inflammation by repressing proinflammatory genes through the GR signaling pathway [262,263]. A single-blind, randomized, controlled clinical trial (IRCT20200404046947N1) was conducted in Iran in patients with severe COVID-19 with early lung damage. The first group was administered 250 mg of intravenous methylprednisolone once daily for three days plus standard care (34 patients), while the control group only received standard care (34 patients). Patients administered methylprednisolone had a statistically significantly lower mortality rate, compared to the control group (5.9% vs. 42.9%, *p* < 0.001) [264].

In a randomized, open-label, multi-center clinical trial (NCT04780581) considering patients with severe COVID-19, 35 patients were administered 40 mg (once daily for three days) or 20 mg (once daily for three days) of methylprednisolone, and were compared to a group of 29 patients who received standard care. Significant differences were observed in favor of the methylprednisolone group in the per-protocol analysis on the composite variable (consisting of death, ICU admission, and non-invasive ventilation) [265]. In a Phase 2b, double-blind, placebo-controlled, randomized clinical trial (NCT04343729) conducted on patients hospitalized for COVID-19 in Brazil, one group received 0.5 mg/kg of intravenous methylprednisolone twice daily for five days (194 patients), while the placebo group received saline solution (199 patients). At day 28, there was no significant difference in mortality between the groups. However, people over 60 years who received methylprednisolone had a lower mortality rate than those in the placebo group [266]. The efficacy of the corticosteroids dexamethasone and methylprednisolone in COVID-19 has raised the question of which corticosteroid is better as a treatment, and recent clinical trials have attempted to answer this question.

In three clinical trials carried out in Colombia (ISRCTN33037282), Iran, and Turkey, methylprednisolone (dose of 250 mg per day or 2 mg/kg body weight) presented better results regarding clinical improvement in patients hospitalized for severe COVID-19 than dexamethasone (dose of 6 mg per day), with respect to hospitalization time, ICU admission, hospital stay in ICU, and biomarkers of severity (CRP, dimer D, and LDH) [267,268,269].

### 9.3. Criteria to Consider for Corticosteroids

Corticosteroids are one of the main drugs proposed for treatment of severe COVID-19, and positive results have been reported for dexamethasone and methylprednisolone in terms of decreased mortality rate, hospital stay, ICU requirement, and time. Better results have been observed for methylprednisolone compared to dexamethasone; however, the results for both drugs are dose-dependent, and high doses tend to increase the number of adverse effects. A meta-analysis considering 96,852 patients reported that the use of corticosteroids increased the mortality of patients with COVID-19 [270], which could be due to the early use of corticosteroids facilitating the viral replication of SARS-CoV-2 due to low immune resistance, leading to an increase in viral load. Therefore, the WHO only recommends their use in critically ill COVID-19 patients at 7 days after the first symptom [271,272]. Hence, the use of this drug will depend on the timing and characteristics of each patient, in order to obtain a positive prognosis. In addition, the use of normal doses of corticosteroids may not benefit COVID-19 patients with renal pathologies or with renal replacement therapy, and should only be used as an option in routine follow-up of the patient, in order to avoid complications [244]. In pregnant women and their fetuses, the treatments appear to have no adverse effects; however, according to pharmacokinetics studies, the clearance of corticosteroids in women is higher. Thus, it may be necessary to modify the recommended doses. In addition, adverse effects on the fetus have been reported with the use of dexamethasone, such as congenital malformations; however, most of the reported cases involved the use of teratogenic drugs and, so, the results cannot be fully attributed to dexamethasone [245].

## 10. Protease Inhibitors

The SARS-CoV-2 viral cycle begins with entry of the genetic material of the virion into the host cell through two different mechanisms: fusion with the plasma membrane or endocytosis. In either case, proteases are required for viral infection, such as angiotensin-converting enzyme 2 (ACE2), which interacts with viral protein S, as well as furin cleavage and superficial serine two transmembrane protease (TMPRSS2), which are key to membrane fusion and L-cathepsin by endocytosis [273,274]. After the virion releases the viral genome into the host cell cytoplasm, translation of ORF1a and ORF1b expresses the pp1a and pp1ab polyproteins, which are cleaved by viral and host cell proteases to yield proteins that form the RdRp complex [209,210,211]. As proteases are essential in SARS-CoV-2 infection and RdRp formation, protease inhibitor drugs are being developed and repurposed, with assessment of their performance through clinical trials (see Table 4).

### 10.1. Nirmatrelvir/Ritonavir

Nirmatrelvir/ritonavir (Paxlovid) is an antiviral protease inhibitor drug developed by Pfizer. Nirmatrelvir (PF-07321332) inhibits the viral protease Mpro (conserved in coronaviruses), which cleaves viral polypeptides (PP1a/PP1ab) responsible for the production of nsp’s that make up the RdRp for viral replication. Ritonavir (a drug used against HIV) inhibits cytochrome P450 (CYP3A4) and increases the half-life of nirmatrelvir in the patient [279,280,281]. In a Phase 1, double-blind, randomized, controlled clinical trial, it was found that nirmatrelvir (with and without ritonavir) is safe and tolerable and, when used in conjunction with ritonavir, the half-life and maximum concentration of nirmatrelvir are increased. Consequently, the dose selected for Phase 2/3 clinical trials was 300 mg nirmatrelvir and 100 mg ritonavir [282].

In a randomized, controlled, double-blind, phase 2/3 clinical trial in unvaccinated patients with mild COVID-19 at high risk of progression to severe COVID-19, 1120 patients were treated with nirmatrelvir/ritonavir (300/100 mg every 12 h for five days), compared to a control group of 1126 patients who received placebo. The main result was that nirmatrelvir/ritonavir decreased the risk of severe disease progression by 89%, leading to lower mortality (0 N/R vs. 13 placebo) and lower viral load during the 14 days of the study and at day 5, with a significant difference compared to placebo; furthermore, there was no significant difference in adverse events (22.6% N/R vs. 23.9% placebo) [275].

In a retrospective cohort study in patients at high risk of severe COVID-19, Paxlovid was administered to 4737 patients, compared with 135,482 patients presenting a vaccination schedule against SARS-CoV-2. Both treatments presented good HR (0.54, 0.39–0.75; 0.20, 0.17–0.22, respectively), and Paxlovid had significantly higher efficacy in patients with risk factors (*p* < 0.05), such as age (over 60 years), cardiovascular disease or neurodegenerative diseases, and immunocompromised patients [283].

In a meta-analysis of clinical trials of oral antiviral drugs (i.e., molnupiravir, fluvoxamine, and Paxlovid), it was determined that the use of these antivirals reduced the mortality rate by 67% (OR = 0.33, 0.22–0.49), with an OR for mortality in the placebo group of 0.41 (0.26–0.64), and an OR in the Paxlovid united group of 0.05 (0.00–0.81) [284].

This review was based on clinical trials; however, it is essential to mention two in vitro studies in Vero-E6 cells infected with SARS-CoV-2 variants (alpha, beta, gamma, delta, omicron, and wild-type). One of them demonstrated the efficacy of nirmatrelvir against any of the variants, according to the 50% effective concentration (EC50) of cell-induced fluorescence recovery [285]; while the other analyzed the kinetics of the Mpro of the SARS-CoV-2 variants and identified that, at a concentration of 50 nM, there was greater than 50% inhibition, while the concentration of 100 nM caused 100% inhibition in all variants [286].

Nirmatrelvir/ritonavir is emerging as the first treatment with high efficacy for mild to moderate COVID-19. On 22 December 2021, the FDA cleared it for use in mild to moderate COVID-19 in adult or pediatric patients at high risk of progression to severe COVID-19 (last updated 14 April 2022). In the United Kingdom, approval was granted on 31 December 2021, for adults who do not require supplemental oxygen and are at risk of progression to severe COVID-19, and approval was granted on 28 January 2022 by the European Medicines Agency. However, further clinical trials are required before it can be used as a treatment for severe COVID-19.

### 10.2. Lopinavir/Ritonavir

Lopinavir and ritonavir are protease inhibitor drugs used as a treatment for human immunodeficiency virus (HIV), which are used in combination to increase the bioavailability of lopinavir in the blood (ritonavir inhibits cytochrome P450 3A4, which metabolizes lopinavir). Lopinavir/ritonavir has been identified as inhibiting the 3C-like protease (3CLpro) involved in the cleavage of polypeptides required for the SARS-CoV viral replication complex, as the sequence of the catalytic site of the protease is conserved in coronaviruses. Thus, lopinavir/ritonavir has been used as a potential drug against SARS-CoV-2 [287,288,289]. In an open-label, randomized, controlled trial called “RECOVERY” involving 5040 patients hospitalized for COVID-19, two groups were compared: 1616 patients receiving lopinavir/ritonavir (400 mg and 100 mg for ten days every 12 h, respectively) and 3424 receiving standard care. The results indicated no statistical significance between the two groups for mortality (23% lopinavir/ritonavir vs. 22% standard care), hospital discharge (69% lopinavir/ritonavir vs. 70% standard care), or progression to invasive mechanical ventilation (29% lopinavir/ritonavir vs. 27% standard care) [290].

In Wuhan, China, a randomized, open-label controlled clinical trial was conducted in 199 patients with severe COVID-19 (SaO2: <94%), where 99 of the patients were administered lopinavir/ritonavir (400 and 100 mg twice daily for 14 days, respectively) and the control group (100 patients) received standard care. The lopinavir/ritonavir group presented no significant benefit in time to clinical improvement (clinical improvement index: 1.24 vs. 1.72), viral clearance, mortality (19.2% vs. 25%), and gastrointestinal effects, although a lower incidence of severe complications was observed [291]. Furthermore, in two clinical trials considering severe COVID-19 patients—an international study and the “DisCoVeRy” trial of the European population—the administration of lopinavir/ritonavir was compared with other drugs, such as hydroxychloroquine, IFN-β-1a (only in the international trial), and combination therapy. Both of these studies did not find significant differences regarding SARS-CoV-2 viral clearance, clinical status, and mortality in any of the treatments [292,293]. The results reported in clinical trials on lopinavir/ritonavir generally do not support the use of these drugs for the treatment of COVID-19; however, it is one of the most commonly used COVID-19 treatments.

### 10.3. Teicoplanin

Teicoplanin is a lipoglycopeptide used as an antibiotic against Gram-positive bacteria. Its use as a therapeutic in the context of COVID-19 arose from previous research indicating it as an inhibitor of Ebola, influenza, hepatitis C infections, SARS-CoV, and MERS-CoV, as it can inhibit the protease cathepsin L, thus preventing the release of the viral genome by the late endosome and inhibiting the key cysteine protease (3CL Pro) in the cleavage of functional polypeptides for viral replication [294,295].

At hospital admission, 21 patients in the intensive care unit (ICU) for severe COVID-19 were administered teicoplanin (6 mg/kg every 24 h; 3 loading doses at admission every 12 h) for approximately ten days (range 7–12 days). The patients showed a statistically significant decrease in lymphocyte count, C-reactive protein (CRP), and procalcitonin at 12-day follow-up; 23.8% of patients were weaned from mechanical ventilation; and the ICU case fatality rate was 42.9% [296]. Based on previous results, Ceccarelli et al. conducted a retrospective, observational, and multi-center study in 55 patients with severe COVID-19 hospitalized in the ICU, called the “Tei-COVID study”. Thirty-four patients were treated with teicoplanin (6 mg/kg every 24 h; 3 loading doses at admission every 12 h), while 21 patients received standard care. They observed a significant decrease in CRP in the treatment group and a lower percentage of mortality, compared to the control group (35.2% Teicoplanin vs. 42.8% control) [297].

A retrospective study performed in Turkey in 115 patients hospitalized for COVID-19 divided patients into two groups: the first was treated with an initial load of 800 mg of teicoplanin and 400 mg at 24 h (54 patients), while the second group was treated with standard care (61 patients). They observed a statistically significant difference in the mortality rates of the groups, at 1.9% (1/54) in the patients who received teicoplanin and 14.8% (9/61) in the standard care group [295]. The use of teicoplanin as first-line treatment is promising, according to clinical trials considering severe COVID-19, as it may significantly decrease the mortality rate. However, the development of randomized and standardized clinical trials is required to obtain results with greater certainty and validate the results obtained so far.

### 10.4. Camostat Mesylate

Camostat mesylate is a drug licensed to treat chronic pancreatitis and post-operative reflux esophagitis, with mild side-effects at high doses (900 mg: urticaria and edema). Notably, it can inhibit transmembrane proteases, including trans-membrane protease serine-2 (TMPRSS-2), TMPRSS-13, and TMPRSS-11D/E/F. Once administered, this drug is metabolized by hydrolysis of its ester group, producing 4-(4-guanidinobenzoyl-oxy) phenylacetic acid (GBPA), with a half-life of one hour in terms of its protease inhibitory potential [298,299,300].

In another double-blind, randomized, multi-center clinical trial in patients hospitalized for COVID-19, 137 patients were administered 200 mg of camostat (three times a day for five days) and 68 patients received a placebo. The results obtained indicated no differences between the two groups regarding recovery time, mortality, and frequency of adverse effects [299]. In contrast, a retrospective study in 371 patients from the United Arab Emirates showed a shorter hospital stay in the ICU (9 days vs. 18 days) and a lower ICU/hospital mortality rate (9.9% vs. 26.5%) [301]. The diversity of clinical trial results suggests that further clinical trials are necessary to determine the usefulness of camostat methylates for the treatment of COVID-19. However, the clinical trial conducted in the Arab Emirates shows promise for its use in severe COVID-19 cases.

### 10.5. Alpha-1-Antitrypsin

Alpha-1-antitrypsin (AAT) is a 52 kDa glycoprotein synthesized in the liver with high concentrations at plasma level, which possesses anti-inflammatory functions (inhibition of NF-kB, IL-8), as well as inhibiting serine proteases such as metalloproteinase 17 (ADAM17) and transmembrane surface serine protease 2 (TMPRSS2), which are crucial for entry of SARS-CoV-2 into the host cell and associated with poor disease prognosis, It is found at low concentrations in patients with severe COVID-19 [302,303,304,305]. In a pre-clinical in vitro assay with HEK293T, Caco2, Vero E6, and human small airway epithelial cells (SAEC) cell lines, AAT was observed to directly inhibit TMPRSS2 by suppressing viral replication in the cell lines (83% decrease in viral titers in SAEC) at concentrations of 40–45 µM (similar to physiological concentrations of between 10–40 µM in alveolar interstitial fluid) [306]. In an assay using two- and three-dimensional organoid cultures, it was found that AAT inhibits TMPRSS2 at concentrations of 1 and 5 mg/mL in undifferentiated epithelial cells; in Calu-3 (human lung) cells, it was determined that, at concentrations of 10 mg/mL, 50% of SARS-CoV-2 viral titers were reduced. Based on the results, a clinical trial was conducted in 9 patients, who were administered inhaled AAT (100 mg for 7 days) or inhaled/intravenous AAT (60 mg/kg body weight on days 1, 3, and 5). Considering the results of the clinical trial, AAT treatment was considered to be safe and effective, improving the initial respiratory status and leading to a lower C-reactive protein (CRP) concentration [307]. AAT is a promising treatment for COVID-19, as it is an FDA-approved drug for the treatment of AAT deficiency. Further clinical trial results will be key in determining its efficacy in the context of SARS-CoV-2 infection.

### 10.6. Criteria to Consider for Protease Inhibitors

Protease inhibitors are drugs with indirect but highly effective mechanisms, as they inhibit the proteases necessary for SARS-CoV-2 replication. Nirmatrelvir/ritonavir is one of the most promising drugs for patients with severe COVID-19; although clinical trials conducted in patients with mild COVID-19 presented risk factors, it decreases progression to severe COVID-19 and increases viral clearance. However, nirmatrelvir/ritonavir may interact with treatments in cancer patients, as the inhibition of CYP450 by ritonavir increases plasma levels of the metabolites of cancer treatments, with their reduced excretion increasing their side-effects [252,308]. Their use is not recommended in patients with renal insufficiency (e.g., an eGFR of less than 30 mL/min) [309]. Teicoplanin and alpha 1-antitrypsin are poorly studied drugs in the context of COVID-19, but they have shown positive results in severe COVID-19, as they have been reported to decrease the mortality rate and CRP concentration. Conflicting results have been reported for camostat mesylate, with some clinical trials reporting a decrease in hospital stay and mortality rate, while others reported no difference from standard care. Camostat mesylate, teicoplanin, and alpha 1-antitrypsin are considered safe drugs, with only mild side-effects having been reported, including skin rash, pruritus, headache, nausea, abdominal pain, and elevated liver enzymes [298,310,311]. Lopinavir is one of the drugs that was believed to work against COVID-19; however, in severe COVID-19 cases, it did not show any positive results when compared to standard care. Furthermore, although adverse effects have not been reported in pregnant women, it is not recommended for use in oral solutions, as they typically contain alcohol and propylene glycol [248,312].

## 11. Others

### 11.1. Heparin

Heparin is a long and linear highly sulfated heparan sulfate (HS) glycosamino-glycan purified from porcine intestines. Its sulfated nature confers heparin with the highest negative charge density of any known biomolecule, which allows heparin to strongly and selectively interact with an immense number of proteins, the most classic being its interaction with serine protease inhibitor antithrombin-III (AT3), facilitating its anticoagulant activity, which is dependent on the presence of a precise penta-saccharide sequence within longer HS chains that allows for AT3 binding [313]. Many severe cases of COVID-19 lead to the development of a clinically significant coagulopathy that is characterized by thrombocytopenia, minor prolongation of prothrombin time (PT) and partial thromboplastin time (aPTT), and elevated serum D-dimer and fibrinogen levels [314]. In severe cases of COVID-19, heparin has been shown to reduce mortality at both prophylactic and therapeutic dosages; however, an increase in bleeding has also been reported. As demonstrated in several randomized clinical trials, heparin plays a central role in COVID-19 treatment and may also have a role in the prevention of post-discharge COVID-19 sequelae in the presence of high-risk clinical features that increase the risk of thrombotic complications [315,316,317].

### 11.2. Colchicine

Colchicine is a drug that has been used for a long time to effectively treat gout, calcium pyrophosphate deposition disease (CPPD), and familial Mediterranean fever (MMF), as well as for the prevention of pericarditis, atrial fibrillation, and myocardial infarction [318,319]. The efficacy of colchicine treatment against COVID-19 has been evaluated due to its potent anti-inflammatory properties, including suppression of the excessive function of neutrophils, monocytes, and macrophages; inhibition of the activation of the inflammasome (mediated in viroporin E in SARS-CoV-2 infection); and the consequent impairment of the IL-1β production. Abolishment of the secretion of IL-6 and TNF-α has also been reported [320,321]. Colchicine can reduce the production of ROS and α-defensin and inhibit neutrophil–platelet aggregation; it has anti-thrombotic, anti-fibrotic, and cardioprotective properties, all factors with special importance in the context of COVID-19 [322,323,324]. Despite its potential efficacy as validated in other pathologies, clinical trials in COVID-19 patients (NCT04367168, NCT04818489) have demonstrated that colchicine was not effective in preventing progression to critical disease or death [325,326].

## 12. Conclusions

From the current perspective and with the information available derived from the various clinical trials focused on potential treatments against COVID-19, we suggest that it is necessary to standardize certain variables to facilitate verification of the efficacy of such treatments, such as the viral clearance time, biomarkers associated with severity, hospital stay, requirement of invasive mechanical ventilation, and mortality rate. One of the main difficulties when comparing results is the large number of variables that need to be normalized, as is the case with multiple treatments applied in a cocktail, which could mask the result—either enhancing or decreasing the desired effect. For example, steroid therapy (considered a conventional treatment), through its extensive pleiotropic effects, could compromise the expected results of a secondary drug. Considering this, a rigorous criterion must be applied to validate the data, attempting to match the conditions of previous studies as much as possible in order to corroborate successful results. In the majority of potential and promising treatment cases, there remain certain unknowns, such as the most efficient dose, the frequency, the time of application throughout the disease, and possible synergies of the drug with other compounds or inhibitions. Furthermore, the genetic component should not be discarded when validating the effectiveness of possible therapies. There is still a long way to go, but none of the options should be ruled out if they have presented any benefit in previous studies, whatever the mechanism used for viral restriction, as there is still no drug on the market with proven high effectiveness against COVID-19.

We also recommend not discarding therapeutic options that previously showed any effectiveness against any previous variant but have decreased effectiveness against new variants of concern because, as mutations appear in the future, these options may recover their effectiveness. According to the Coronavirus drug and treatment tracker [327], the most-applied clinical drugs in the U.S. against COVID-19 up to August 2022 were Paxlovid, Remdesivir, Molnupiravir, Evusheld (tixagevimab and cilgavimab), Bebtelovimab, Dexamethasone, and Baricitinib. The actual trend to date has primarily focused on the development of and research into antiviral drugs (mainly viral polymerase inhibitors). In second place has been the development of more efficient anti-inflammatories and, in third place, the development of mAb cocktails capable of neutralizing a wide spectrum of emerging variants. Due to the role played by viral polymerase inhibitor antivirals accompanied by anti-inflammatory therapeutics during the pandemic, if research efforts are focused on the development of broad-spectrum viral polymerase inhibitor drugs, remarkable results may be obtained that could be further extrapolated to other viral pathologies.

## Figures and Tables

**Table 1:** Anti-Spike mAbs tested in clinical trials.

mAbs	Source	Admin	Phase	Identification Register	Use	Main Sponsor	Cite
casirivimab and imdevimab	Recombinant Human/Humanized mice	Cocktail	1/2/3	NCT04425629	Ambulatory	Regeneron, Greenburgh, NY, USA	[54]
casirivimab and imdevimab	Recombinant Human/Humanized mice	Cocktail	1/2/3	NCT04426695	Hospitalized patients	Regeneron	[74]
casirivimab and imdevimab	Recombinant Human/Humanized mice	Cocktail	3	NCT04452318	Prophylaxis	Regeneron	[75]
MW33	Recombinant	Monotherapy	1	NCT04533048	Ambulatory	Mabwell (Shanghai) Bioscience Co., Ltd., Shanghai, China	[76]
MW33	Recombinant	Monotherapy	2	NCT04627584	Ambulatory	Mabwell (Shanghai) Bioscience Co., Ltd.	[76]
BRII-196 and BRII-198	Recombinant Human	Cocktail	2/3	NCT05780424	Ambulatory	Eli Lilly, Indianapolis, IN, USA	[77]
BRII-196	Recombinant Human	Monotherapy	1	NCT04479631	Ambulatory	Brii Biosciences Limited, Beijing, China	[18]
BRII-198	Recombinant Human	Monotherapy	1	NCT04479644	Ambulatory	Brii Biosciences Limited	[18]
AZD8895 and AZD1061	Recombinant Human	Cocktail	3	NCT04625725	Ambulatory	AstraZeneca, Cambridge, UK	[78]
XAV-19	Polyclonal Humanized	Monotherapy	2	NCT04453384	Hospitalized patients	Nantes University Hospital, Nantes, France	[79]
XAV-19	Polyclonal Humanized	Monotherapy	2/3	NCT04928430	Hospitalized patients	Xenothera SAS, Nantes, France	[79]
Sotrovimab	Recombinant Human	Monotherapy	1	NCT04988152	Ambulatory	Vir Biotechnology, Inc., San Francisco, CA, USA	[80]
Sotrovimab	Recombinant Human	Monotherapy	2/3	NCT04545060	Ambulatory	Vir Biotechnology, Inc.	[81]
Regdanvimab	Recombinant Human	Monotherapy	1	NCT04593641	Ambulatory	Celltrion, Incheon, Republic of Korea	[82]
Regdanvimab	Recombinant Human	Monotherapy	2/3	NCT04602000	Ambulatory	Celltrion	[82]

**Table 2:** Viral polymerase inhibitors against SARS-CoV-2 tested in clinical trials.

Drug	Doses	Comparator	Phase	Identification Register	Use	Main Sponsor	Cite
Remdesivir	One dose of 200 mg on day 1, followed by doses of 100 mg iv once a day for nine days	Remdesivir placebo	3	NCT04257656	Hospitalized Patients	China-Japan Friendship Hospital	[212]
Ribavirin	Oral Lopinavir/ritonavir: 400 mg/100 mg twice daily for 14 days; oral Ribavirin 400 mg twice daily for 14 days; Interferon Beta-1B 0.25 mg subcutaneous injection alternate days for three days	Lopinavir/ritonavir	2	NCT04276688	Hospitalized Patients	The University of Hong Kong	[213]
Favipiravir	Oral avipiravir 1600 mg stat and then 600 mg every 8 h plus hydroxychloroquine 200 mg twice a day for 1 week	Lopinavir/Ritonavir and hydroxychloroquine	3	IRCT20151227025726N14	Patients with moderate to severe COVID-19	Royal College of Surgeons in Ireland—Medical University of Bahrain	[214]
Molnupiravir	Oral doses of 200/400/800 mg every 12 h for five days	Placebo	2/3	NCT04575584	Hospitalized Patients	Merck Sharp and Dohme Corp	[215]
Sofosbuvir/ Daclastavir	Oral Sofosbuvir 400 mg plus Daclatasvir 200 mg for day	Standard clinical care	3	NCT04535869	Patients with Moderate to severe COVID-19	Mansoura University	[216]

**Table 3:** Corticosteroids in clinical trials for COVID-19.

Drug	Doses	Comparator	Phase	Identification Register	Use	Main Sponsor	Cite
Dexamethasone	20 mg/iv/daily/from Day 1 of randomization during 5 days, followed by 10 mg/iv/daily from Day 6 to 10 of randomization	Standard clinical care	4	NCT04325061	Patients intubated and mechanically ventilated	Dr. Negrin University Hospital	[253]
Methylprednisolone	1 mg/kg/day given intravenously for five days	Dexamethasone	3	NCT04603729	Patients with moderate to severe COVID-19	Fatima Memorial Hospital	[254]

**Table 4:** Protease inhibitors in clinical trials.

Drug	Doses	Comparator	Phase	Identification Register	Use	Main Sponsor	Cite
Nirmatrelvir/ Ritonavir	300/100 mg every 12 h for 5 days	Placebo	3	NCT04960202	Ambulatory	Pfizer	[275]
Lopinavir/ Ritonavir	200 mg/100 mg 2 tablets by mouth, every 12 h for 7–10 days	Hydroxychloroquine	2	NCT04307693	Ambulatory	Asan Medical Center	[276]
Camostat mesylate	200 mg taken orally, four times daily, for seven days	Placebo	2	NCT04353284	Ambulatory	Yale University	[277]
Alpha-1-antitrypsin	Administration based on clinical indication	No comparator	No records	NCT04799873	Patients without the need for invasive ventilation	Universität des Saarlandes	[278]

## Data Availability

Not applicable.
